# Clustering microbiome data using mixtures of logistic normal multinomial models

**DOI:** 10.1038/s41598-023-41318-8

**Published:** 2023-09-07

**Authors:** Yuan Fang, Sanjeena Subedi

**Affiliations:** 1 School of Pharmacy and Pharmaceutical Sciences, Binghamton University, State University of New York, 4400 Vestal Parkway East, Binghamton, NY 13902 USA; 2 School of Mathematics and Statistics, 4302 Herzberg Laboratories, Carleton University, 1125 Colonel By Drive, Ottawa, ON K1S 5B6 Canada

**Keywords:** Statistics, Statistical methods

## Abstract

Discrete data such as counts of microbiome taxa resulting from next-generation sequencing are routinely encountered in bioinformatics. Taxa count data in microbiome studies are typically high-dimensional, over-dispersed, and can only reveal relative abundance therefore being treated as compositional. Analyzing compositional data presents many challenges because they are restricted to a simplex. In a logistic normal multinomial model, the relative abundance is mapped from a simplex to a latent variable that exists on the real Euclidean space using the additive log-ratio transformation. While a logistic normal multinomial approach brings flexibility for modeling the data, it comes with a heavy computational cost as the parameter estimation typically relies on Bayesian techniques. In this paper, we develop a novel mixture of logistic normal multinomial models for clustering microbiome data. Additionally, we utilize an efficient framework for parameter estimation using variational Gaussian approximations (VGA). Adopting a variational Gaussian approximation for the posterior of the latent variable reduces the computational overhead substantially. The proposed method is illustrated on simulated and real datasets.

## Introduction

The human microbiome comprises complex communities of microorganisms including but not limited to bacteria, fungi, and viruses, that inhabit in and on a human body^1,2^. It is estimated that there are approximately $10^{14}$ microbial cells associated with the human body, which is around 10 times the number of human cells^3,4^. The human microbiome plays a significant role in human health and disease status. There is evidence indicating that microbial dysbiosis may lead to diseases such as cardiovascular diseases^5^, diabetes^6^, inflammatory bowel disease^7^, obesity^8^, and many others. Next-generation sequencing techniques, such as the 16S ribosomal RNA (rRNA) amplicon sequencing or shotgun metagenomics sequencing, provide an effective way for quantification and comparison of the bacterial composition, including types and abundance of different bacteria within biological samples^9–12^. In 16S rRNA sequencing, the 16S rRNA, which is ubiquitous in all bacterial organisms but also has distinct variable regions that can be used to discriminate between different bacteria is first PCR-amplified and then sequenced^10^. Shotgun sequencing on the other hand is an untargeted sequencing of all microbial genomes in a sample^13^. In either case, short reads are preprocessed through steps of quality control and filtering steps. The processed raw sequence reads are then clustered into operational taxonomic units (OTUs) at a certain similarity level^14^ where each OTU is characterized by a representative DNA sequence that could be assigned to a taxonomic lineage by comparing to a known database^2^. Resulting read counts at different taxonomic levels for *n* samples over $K+1$ taxa are stored as a $n\times (K+1)$ matrix $\textbf{W}$, with the entry *W*[*i*, *k*] representing the counts recorded for the $k^{th}$ taxon in the $i^{th}$ sample.

Statistical analysis of microbiome data is complicated. The microbiome count data can only reveal relative abundance, i.e., the abundance for each taxa is constrained by the total sum of the microbes in that particular sample and the total sum of microbes could vary among the samples depending on the sequencing depth. Different individuals could share various communities of microorganisms, with only a few major ones in common, and even for one person, the microbial composition could be totally different in different body sites. The heterogeneity of the microbiome samples also leads to over-dispersion. See Hamady and Knight^15^ for a detailed review of challenges related to analyzing microbiome data. Standard multivariate analysis usually fails to capture these properties of the microbiome data. Different models have been proposed for the microbiome counts in the literature that captures one or more of the above intrinsic characteristics such as the negative binomial model^16^, zero-inflated negative binomial model^17^, zero-inflated Poisson model^18,19^, Dirichlet-multinomial model^20–22^, and the logistic normal multinomial model^23^. While modelling such count data, a negative binomial (NB) model can allow for the variance to be larger than the mean using a dispersion parameter, thus handling over-dispersion better than a simple Poison model. The zero-inflated negative binomial (ZINB) and zero-inflated Poisson (ZIP) have been proposed to account for the excessive number of zeros^18^. Xu et al.^24^ provides a comparison among the zero-inflated models. However, the NB and ZINB models ignore the compositional nature of these microbial counts. Chen and Li^21^, Holmes et al.^20^, Wadsworth et al.^25^, and Subedi et al.^22^ utilized the Dirichlet-multinomial model for microbial counts that takes into account the compositional nature of these data. Alternately, Xia et al.^23^ employed the logistic normal multinomial model, mapping the relative abundance from a simplex to a latent variable that exists on the real Euclidian space using the additive log-ratio transformation. Cao et al.^26^ exploited a Poisson-multinomial model and performed a multi-sample estimation of microbial composition in positive simplex space from a high-dimensional sparse count table. Caporaso et al.^27^ quantified variations of microbial composition across time by projecting the dynamics using low-dimensional embedding. Äijö et al.^12^ proposed a temporal probabilistic model for the microbiome composition using a hierarchical multinomial model. Silverman et al.^28^ also developed a dynamic linear model based on the logistic normal multinomial model to study the artificial human guts microbiome.

Clustering microbiome samples into groups that share similar microbial compositional patterns is of great interest^20^. Clustering algorithms are usually categorized into hierarchical clustering and distance-based clustering. Hierarchical clustering has been applied for clustering microbiome data, yet it requires the choice of a cut-off threshold, according to which samples can be divided into groups^20^. On the other hand, $k-$means clustering, a distance-based method, might not be appropriate for microbiome compositions because it is typically used for continuous data and obtains spherical clusters. Hence, model-based clustering approaches that utilize a finite mixture model have been widely used in the last decade to cluster microbiome data^20,22^. A finite mixture model assumes that the population consists of a finite mixture of subpopulations (or clusters), each represented by a known distribution^29–32^. Due to the flexibility in choosing component distributions to model different types of data, several mixture models based on discrete distributions have been developed to study count data, especially, for gene expression data. Rau et al.^33^ proposed a clustering approach for RNA-seq data using mixtures of univariate Poisson distributions; Papastamoulis et al.^34^ proposed a mixture of Poisson regression models; Si et al.^35^ studied model-based clustering for RNA-seq data using a mixture of negative binomial (NB) distributions; Silva et al.^36^ proposed a multivariate Poisson-log normal mixture model for clustering gene expression data. However, due to the compositional nature of microbiome data, none of the above discrete mixture models can be employed directly for clustering microbiome data. Holmes et al.^20^ adopted the Dirichlet-multinomial (DM) model, where the underlying compositions are modeled as a Dirichlet prior to a multinomial distribution that describes the taxa counts, and proposed a mixture of DM models to cluster samples.

In this paper, we develop a model-based clustering approach using the logistic normal multinomial model proposed by Xia et al^23^ to cluster microbiome data. In the logistic normal multinomial model, the observed counts are modeled using a multinomial distribution, and the relative abundance is regarded as a random vector on a simplex, which is further mapped to a latent variable that exists on the real Euclidean space through an additive log-ratio transformation. While this approach captures the additional variability compared to a multinomial model, it does not possess a closed form expression of the log-likelihood functions and of the posterior distributions of the latent variables. Therefore, the expected complete-data log-likelihoods needed in the E-step of a traditional EM algorithm are usually intractable. In such a scenario, one commonly used approach is a variant of the EM algorithm that relies on Bayesian techniques using Markov chain Monte Carlo (MCMC); however, this would typically bring in high computational cost. Here, we develop a variant of the EM algorithm, here on referred to as a variational EM algorithm for parameter estimation that utilizes variational Gaussian approximations (VGA). In Variational Gaussian approximations (VGA)^37^, a complex posterior distribution is approximated using computationally convenient Gaussian densities by minimizing the Kullback-Leibler (KL) divergence between the true and the approximating densities^38,39^. Adopting a variational Gaussian approximation delivers accurate approximations of the complex posterior while reducing computational overhead substantially. Hence, this approach has become extremely popular in many different fields of machine learning^37,38,40–43^.

The contribution of the paper is two folds - first, we develop a computationally efficient framework for parameter estimation for a logistic normal multinomial model through the use of variational Gaussian approximations and second, we utilize this framework to develop a model-based clustering framework for clustering microbiome data. Through simulations and applications to microbiome data, the utilities of the proposed approach are illustrated. The paper is structured as follows: First two subsections in the Methods section describe the logistic normal multinomial model for microbiome count data and detail the variational Gaussian approximations. The third and fourth subsections in the Methods section provide a mixture model framework based on the model described above together with a variational EM algorithm for parameter estimation. In the Results section, clustering results are illustrated by applying the proposed algorithm to both simulated and real data. Finally, a discussion on the advantages and limitations along with some future directions are provided in the Discussion section.

## Methods

### The logistic normal multinomial model for microbiome compositional data

Suppose we have $K+1$ bacterial taxa for a sample denoted as a random vector ${\textbf{W}}= (W_1,\dots ,W_{K+1})^\top$. Here, the taxa could represent any level of the bacterial phylogeny such as OTU, species, genus, phylum, etc. Due to the fact that taxa count from 16S sequencing can only reveal relative abundance, let’s suppose there is a vector ${\varvec{\Theta }}= (\Theta _,\dots ,\Theta _{K+1})$ such that $\sum _{k=1}^{K+1}{\Theta _k} = 1$, which represents the underlying composition of the bacterial taxa. Then, the microbial taxa count ${\textbf{W}}$ can be modeled as a multinomial random variable with the following conditional density function:

$$\begin{aligned} p({\textbf{w}}|{\varvec{\Theta }}) \propto \prod _{k=1}^{K+1}(\Theta _k)^{w_k}. \end{aligned}$$

Several models have been proposed in the literature that capture the relative abundance nature of microbiome data and analyze the compositional data^20,23^. Here we use the model by Xia et al.^23^ that utilizes an additive log-ratio (ALR) transformation $\phi ({\varvec{\Theta }})$ proposed by Aitchison^44^ such that:

1 $$\begin{aligned} {\textbf{Y}}= \phi ({\varvec{\Theta }}) = \left( \log \left( \dfrac{\Theta _1}{\Theta _{K+1}}\right) ,\dots ,\log \left( \dfrac{\Theta _K}{\Theta _{K+1}}\right) \right) ^\top . \end{aligned}$$

This transformation $\phi$ maps the vector ${\varvec{\Theta }}$ from a *K*-dimensional simplex to the *K*-dimensional real space ${\mathbb {R}}^K$. The prior distribution for ${\textbf{Y}}$ is assumed to be a multivariate normal distribution with parameters ${\varvec{\mu }}$ and ${\varvec{\Sigma }}$ with the density function

$$\begin{aligned} p({\textbf{y}}|{\varvec{\mu }},{\varvec{\Sigma }}) \propto |{\varvec{\Sigma }}|^{-\frac{1}{2}}\exp \left\{ -\frac{1}{2}({\textbf{y}}-{\varvec{\mu }})^\top {\varvec{\Sigma }}^{-1}({\textbf{y}}-{\varvec{\mu }})\right\} . \end{aligned}$$

As this additive log-ratio transformation is a one-to-one map, the inverse operator of $\phi$ exists and is given by

$$\begin{aligned} {\varvec{\Theta }}= \phi ^{-1}({\textbf{Y}})={\left\{ \begin{array}{ll} \dfrac{\exp (Y_k)}{1+\sum _{k=1}^{K}{\exp (Y_k)}} &{} k=1,\dots ,K \\ \dfrac{1}{1+\sum _{k=1}^{K}{\exp (Y_k)}} &{} k= K+1 \end{array}\right. }. \end{aligned}$$

Hence, the joint density of ${\textbf{W}}$ and ${\textbf{Y}}$ up to a constant is as follows:

$$\begin{aligned} \begin{aligned} p({\textbf{w}},{\textbf{y}})&\propto p\left( {\textbf{w}}|\phi ^{-1}({\textbf{y}})\right) p({\textbf{y}}|{\varvec{\mu }},{\varvec{\Sigma }})=\prod _{k=1}^{K+1}\left( {\phi ^{-1}({\textbf{y}})}_k\right) ^{w_k}\times |{\varvec{\Sigma }}|^{-\frac{1}{2}}\exp \left\{ -\frac{1}{2}({\textbf{y}}-{\varvec{\mu }})^\top {\varvec{\Sigma }}^{-1}({\textbf{y}}-{\varvec{\mu }})\right\} \end{aligned}. \end{aligned}$$

### A variational Gaussian lower bound

For the microbiome data, only the count vector ${\textbf{W}}$ is observed while the latent variable ${\textbf{Y}}$ is unobserved. The marginal density of ${\textbf{W}}$ can be written as

$$\begin{aligned} p({\textbf{w}}) = \int _{{\mathbb {R}}^K} p({\textbf{w}},{\textbf{y}}) d{\textbf{y}}\propto \int _{{\mathbb {R}}^K} \prod _{k=1}^{K+1}\left( {\phi ^{-1}({\textbf{y}})}_k\right) ^{w_k}\times |{\varvec{\Sigma }}|^{-\frac{1}{2}}\exp \left\{ -\frac{1}{2}({\textbf{y}}-{\varvec{\mu }})^\top {\varvec{\Sigma }}^{-1}({\textbf{y}}-{\varvec{\mu }})\right\} d{\textbf{y}}. \end{aligned}$$

Note that this marginal distribution of ${\textbf{W}}$ involves multiple integrals and cannot be further simplified. Here, in the presence of missing data, an expectation-maximization (EM) algorithm^45^ or some variant of it is typically utilized for parameter estimation. An EM algorithm comprises two steps: an E-step in which the expected value of the complete data (i.e. observed and missing data) log-likelihood is computed given the observed data and current parameter estimate and an M-step in which the complete data log-likelihood is maximized. These steps are repeated until convergence to obtain the maximum likelihood estimate of the parameters. To compute the expected value of the complete data log-likelihood, ${\mathbb {E}}({\textbf{Y}}\mid {\textbf{w}})$ and ${\mathbb {E}}({\textbf{Y}}{\textbf{Y}}^T\mid {\textbf{w}})$ needs to be computed for which we need $p({\textbf{y}}|{\textbf{w}})$. Mathematically,

$$\begin{aligned} p({\textbf{y}}|{\textbf{w}})=\frac{p({\textbf{w}},{\textbf{y}})}{p({\textbf{w}})} = \frac{\prod _{k=1}^{K+1}\left( {\phi ^{-1}({\textbf{y}})}_k\right) ^{w_k}\times |{\varvec{\Sigma }}|^{-\frac{1}{2}}\exp \left\{ -\frac{1}{2}({\textbf{y}}-{\varvec{\mu }})^\top {\varvec{\Sigma }}^{-1}({\textbf{y}}-{\varvec{\mu }})\right\} }{\int _{{\mathbb {R}}^K}\prod _{k=1}^{K+1}\left( {\phi ^{-1}({\textbf{y}})}_k\right) ^{w_k}\times |{\varvec{\Sigma }}|^{-\frac{1}{2}}\exp \left\{ -\frac{1}{2}({\textbf{y}}-{\varvec{\mu }})^\top {\varvec{\Sigma }}^{-1}({\textbf{y}}-{\varvec{\mu }})\right\} d{\textbf{y}}}. \end{aligned}$$

However, the denominator involves multiple integrals and cannot be further simplified. One could employ a Markov chain Monte Carlo (MCMC) approach to explore the posterior state space; however, these methods are typically computationally expensive, especially for high-dimensional problems. Here, we propose the use of variational Gaussian approximation (VGA)^37^ for parameter estimation. A VGA aims to find an optimal and tractable approximation that has a Gaussian parametric form to approximate the true complex posterior by minimizing the Kullback-Leibler divergence between the true and the approximating densities. It has been successfully used in many practical applications to overcome this challenge^38–42,46^. In order to utilize VGA, we define a new latent variable ${\varvec{\eta }}$ by transforming ${\textbf{Y}}$ such that

2 $$\begin{aligned} {\varvec{\eta }}= B{\textbf{Y}}, \quad \text { where }B=\begin{pmatrix} 1&{}0&{}\dots &{}0\\ 0&{}1&{}\dots &{}0\\ \vdots &{}\vdots &{}\dots &{}\vdots \\ 0&{}0&{}\dots &{}1\\ 0&{}0&{}\dots &{}0 \end{pmatrix}, \end{aligned}$$

is a $(K+1)\times K$ matrix which takes the form as an identity matrix attached by a row of K zeros. Given that ${\textbf{Y}}\sim \mathop {\text {N}}({\varvec{\mu }},{\varvec{\Sigma }})$, the new latent variable ${\varvec{\eta }}\sim \mathop {\text {N}}(\tilde{{\varvec{\mu }}},\tilde{{\varvec{\Sigma }}})$ where

3 $$\begin{aligned} \tilde{{\varvec{\mu }}} = B{\varvec{\mu }}= ({\varvec{\mu }},0)^\top ; \quad \tilde{{\varvec{\Sigma }}} = B{\varvec{\Sigma }}B^\top = \left( \begin{array}{c|c} {\varvec{\Sigma }}&{}{\textbf{0}}_{K\times 1}\\ \hline {\textbf{0}}_{1\times K}&{}0 \end{array}\right) . \end{aligned}$$

Then, the underlying composition variable ${\varvec{\Theta }}$ can be written as a function of ${\varvec{\eta }}$:

4 $$\begin{aligned} {\varvec{\Theta }}= \tilde{\phi }^{-1}({\varvec{\eta }}) = \dfrac{\exp {\eta _k}}{\sum _{k=1}^{K+1}{\exp {\eta _k}}} k = 1\dots ,K+1. \end{aligned}$$

Suppose we have an approximating density $q({\varvec{\eta }})$, then the marginal log density of ${\textbf{W}}$ can be written as:

$$\begin{aligned} \log p({\textbf{w}})&= \int \log p({\textbf{w}}) ~q({\varvec{\eta }}) ~d{\varvec{\eta }}=\int \log \frac{p({\textbf{w}},{\varvec{\eta }})/q({\varvec{\eta }})}{p({\varvec{\eta }}\mid {\textbf{w}})/q({\varvec{\eta }})}~q({\varvec{\eta }})~d{\varvec{\eta }}\\&= \int \left[ \log ~p({\textbf{w}},{\varvec{\eta }}) -\log q({\varvec{\eta }})\right] ~q({\varvec{\eta }})~d{\varvec{\eta }}+\int \log \dfrac{q({\varvec{\eta }})}{p({\varvec{\eta }}|{\textbf{w}})}~ q({\varvec{\eta }})~ d{\varvec{\eta }}\\&= F(q({\varvec{\eta }}),{\textbf{w}}) +D_{KL}(q||p), \end{aligned}$$

where the first part $F(q({\varvec{\eta }}),{\textbf{w}}) = \int q({\varvec{\eta }})\log \dfrac{p({\textbf{w}},{\varvec{\eta }})}{q({\varvec{\eta }})} d{\varvec{\eta }}$ is called the evidence lower bound (ELBO)^37^ and the second part $D_{KL}(q||p)= \int \log \dfrac{q({\varvec{\eta }})}{p({\varvec{\eta }}|{\textbf{w}})}~ q({\varvec{\eta }})~d{\varvec{\eta }}$ is the Kullback-Leibler divergence from $p({\varvec{\eta }}|{\textbf{w}})$ to $q({\varvec{\eta }})$. Hence, minimizing the Kullback-Leibler divergence is equivalent to maximizing the following evidence lower bound (ELBO). In VGA, we assume $q({\varvec{\eta }})$ is a Gaussian distribution, such that

$$\begin{aligned} q({\varvec{\eta }}) = \mathop {\text {N}}({\varvec{\eta }}|{\textbf{m}},V) \propto |V|^{-\frac{1}{2}}\exp \left\{ -\frac{1}{2}({\varvec{\eta }}-{\textbf{m}})^\top V^{-1}({\varvec{\eta }}-{\textbf{m}})\right\} . \end{aligned}$$

Given the fact that $q({\varvec{\eta }})$ is fully characterized by its mean vector and covariance matrix, the above lower bound is a function of the variational parameters ${\textbf{m}}$ and *V* and we aim to find the optimal set of $({\textbf{m}},V)$ such that it maximizes $F(q({\varvec{\eta }},{\textbf{w}}))$. $F(q({\varvec{\eta }},{\textbf{w}}))$ can be separated into three parts:

$$\begin{aligned} F(q({\varvec{\eta }}),{\textbf{w}}) = F({\textbf{m}},V) = - \int q({\varvec{\eta }})\log q({\varvec{\eta }}) d{\varvec{\eta }}+ \int q({\varvec{\eta }})\log 
p({\varvec{\eta }}) d{\varvec{\eta }}+ \int q({\varvec{\eta }})\log p({\textbf{w}}|{\varvec{\eta }}) d{\varvec{\eta }}. \end{aligned}$$

Up to a constant, the last integral, which is denoted as $\gamma$, in the above decomposition is given as follows:

$$\begin{aligned} \begin{aligned} \gamma&= \int q({\varvec{\eta }})\log p({\textbf{w}}|{\varvec{\eta }}) d{\varvec{\eta }}= {\mathbb {E}}_{q({\varvec{\eta }}|{\textbf{m}},V)}\left[ {\textbf{w}}^\top {\varvec{\eta }}-\sum _{k=1}^{K+1}{w_k\log \left( \sum _{k=1}^{K+1}{\exp \eta _k}\right) }\right] \\&= {\textbf{w}}^\top {\textbf{m}}- \left( \sum _{k=1}^{K+1} w_k\right) {\mathbb {E}}_{q({\varvec{\eta }}|{\textbf{m}},V)}\left[ \log \left( \sum _{k=1}^{K+1}{\exp \eta _k}\right) \right] . \end{aligned} \end{aligned}$$

Similar to Blei and Lafferty^47^, we use an upper bound for the expectation of log sum exponential term with a Taylor expansion,

$$\begin{aligned} {\mathbb {E}}_{q({\varvec{\eta }}|{\textbf{m}},V)}\left[ \log \left( \sum _{k=1}^{K+1}{\exp \eta _k}\right) \right] \le \xi ^{-1}\left\{ \sum _{k=1}^{K+1}{\mathbb {E}}_{q({\varvec{\eta }}|{\textbf{m}},V)}\left[ \exp (\eta _k)\right] \right\} -1+\log (\xi ), \end{aligned}$$

where $\xi \in {\mathbb {R}}$ is introduced as a new variational parameter.

Here, we further assume that *V* is a diagonal matrix with the first *K* diagonal element of *V* as $v_k^2$ and the $K+1^{th}$ diagonal element is set to 0 such that

$$\begin{aligned} v_k^2 = {\left\{ \begin{array}{ll} v_k^2,&{}k=1,\dots ,K\\ 0,&{}k=K+1. \end{array}\right. } \end{aligned}$$

We also denote the $k-$th element of ${\textbf{m}}$ as $m_k$ such that

$$\begin{aligned} m_k = {\left\{ \begin{array}{ll} m_k, &{}k=1,\dots ,K\\ 0,&{}k=K+1. \end{array}\right. } \end{aligned}$$

Hence, the expectation

$$\begin{aligned} {\mathbb {E}}_{q({\varvec{\eta }}|{\textbf{m}},V)}\left[ \exp (\eta _k)\right] = \exp \left( m_k+\dfrac{ v_k^2}{2}\right) , \text { for }k=1,\dots ,K+1. \end{aligned}$$

Based on this upper bound, we obtain a concave lower bound to $\gamma$ and to the ELBO. The new concave variational Gaussian lower bound to the model evidence $\log p({\textbf{w}})$ is given as follows

5 $$\begin{aligned} \begin{aligned} {\tilde{F}}\left( {\textbf{m}},V,\tilde{{\varvec{\mu }}},\tilde{{\varvec{\Sigma }}},\xi \right) =&{\textbf{w}}^\top {\textbf{m}}- \left( \sum _{k=1}^{K+1} w_k\right) \left\{ \xi ^{-1}\left[ \sum _{k=1}^{K+1}\exp \left( m_k+\dfrac{ v_k^2}{2}\right) \right] -1+\log (\xi )\right\} \\&-\frac{1}{2}\log |B^\top \tilde{{\varvec{\Sigma }}} B|-\frac{1}{2}({\textbf{m}}-\tilde{{\varvec{\mu }}})^\top \tilde{{\varvec{\Sigma }}}^{*}( {\textbf{m}}-\tilde{{\varvec{\mu }}})-\frac{1}{2}\mathop {\text {Tr}}(\tilde{{\varvec{\Sigma }}}^{*} V)+\frac{1}{2}\sum _{k=1}^K\log ( v_k^2)+\dfrac{K}{2}, \end{aligned} \end{aligned}$$

where $$\tilde{{\varvec{\Sigma }}}^{*} = \left( \begin{array}{c|c} {\varvec{\Sigma }}^{-1}&{}{\textbf{0}}_{K\times 1}\\ \hline {\textbf{0}}_{1\times K}&{} 0 \end{array}\right)$$ is the generalized inverse of $\tilde{{\varvec{\Sigma }}}$. Details on the derivation of this lower bound can be found in the Supplementary material Mathematical Detail section. Given fixed ${\textbf{w}}$, ${\tilde{{\varvec{\mu }}}}$, and ${\tilde{{\varvec{\Sigma }}}}$, this lower bound only depends on the variational parameter set $({\textbf{m}},V,\xi )$.

Maximization of the lower bound ${\tilde{F}}\left( {\textbf{m}},V,\tilde{{\varvec{\mu }}},\tilde{{\varvec{\Sigma }}},\xi \right)$ with respect to $\xi$ has a closed form solution and is given by

6 $$\begin{aligned} {\hat{\xi }} = \sum _{k=1}^{K+1}\exp \left( m_k+\dfrac{v_k^2}{2}\right) . \end{aligned}$$

However, maximization with respect to ${\textbf{m}}$ and $v_k, k=1,\dots ,K$ do not have analytical solutions. We use Newton’s method to search for roots to the following derivatives:

7 $$\begin{aligned} \dfrac{\partial {\tilde{F}}}{\partial {\textbf{m}}} = {\textbf{w}}- \tilde{{\varvec{\Sigma }}}^{*}( {\textbf{m}}-{\tilde{{\varvec{\mu }}}})-\left( \sum _{k=1}^{K+1} w_k\right) \xi ^{-1}\exp \left( {\textbf{m}}+\dfrac{{\textbf{v}}^2}{2}\right) , \end{aligned}$$

with ${\textbf{v}}^2=(v_1^2,\dots ,v_K^2,0)$ denoting the diagonal element of *V* as a vector; and

8 $$\begin{aligned} \dfrac{\partial {\tilde{F}}}{\partial v_k} = v_k^{-1}- v_k\tilde{{\varvec{\Sigma }}}_{k,k}^{*} - \left( \sum _{k=1}^{K+1} w_k\right) \xi ^{-1}\exp \left( m_k+\dfrac{ v_k^2}{2}\right) v_k. \end{aligned}$$

Details can be found in the Supplementary material Mathematical Detail section.

### Mixture of logistic normal multinomial models

Assume there are *G* subgroups in the population, with $\pi _g$ denoting the mixing weight of the $g-$th component such that $\sum _{g=1}^{G}\pi _g = 1$. Then, a $G-$component finite mixtures logistic normal multinomial models can be written as

$$\begin{aligned} f({\textbf{w}}\mid \varvec{\vartheta })= \sum _{g=1}^G \pi _g f_g({\textbf{w}}\mid \vartheta _g), \end{aligned}$$

where $f_g({\textbf{w}}\mid \varvec{\vartheta }_g)$ represents the density function of the observation ${\textbf{W}}= {\textbf{w}}$, given that ${\textbf{W}}$ comes from the $g-$th component with parameters $\vartheta _g$.

Provided *n* observed counts, ${\textbf{w}}= ({\textbf{w}}_1,\dots ,{\textbf{w}}_n)$ with a transformed underlying the composition ${\textbf{Y}}_i, i= 1,\dots , n$, the likelihood of a $G-$component finite mixture is given as

$$\begin{aligned} \mathscr {L}(\varvec{\vartheta }\mid {\textbf{w}})=\prod _{i=1}^{n}f(\textbf{w}_i\mid \varvec{\vartheta })= \prod _{i=1}^{n}\sum _{g=1}^G \pi _g f_g(\textbf{w}_i\mid \vartheta _g) = \prod _{i=1}^{n}\sum _{g=1}^G \pi _g \int p({\textbf{w}}_i\mid {\textbf{y}}_i)p({\textbf{y}}_i\mid \vartheta _g) d{\textbf{y}}_i. \end{aligned}$$

In clustering, the unobserved component membership is denoted by an indicator variable $z_{ig}, i=1,\dots ,n, g=1,\dots ,G$ that takes the form

$$\begin{aligned} z_{ig} = {\left\{ \begin{array}{ll} 1 &{} \text { if the } i-th \text { observation is from the } g-th \text { group},\\ 0 &{} \text { otherwise}. \end{array}\right. } \end{aligned}$$

Therefore, conditional on $z_{ig}$, we have

$$\begin{aligned} {\textbf{Y}}_i|z_{ig} = 1 \sim \mathop {\text {N}}({\varvec{\mu }}_g,{\varvec{\Sigma }}_g). \end{aligned}$$

In order to utilize the variational approach for parameter estimation, we again define a new latent variable ${\varvec{\eta }}$ such that ${\varvec{\eta }}= B{\textbf{Y}}$ and

$$\begin{aligned} {\varvec{\eta }}_i\mid z_{ig}=1 \sim \mathop {\text {N}}(\tilde{{\varvec{\mu }}}_g,\tilde{{\varvec{\Sigma }}}_g), \quad \text {where}\quad \tilde{{\varvec{\mu }}}_g = B{\varvec{\mu }}_g = ({\varvec{\mu }}_g,0)^\top \quad \text {and}\quad \tilde{{\varvec{\Sigma }}}_g = B{\varvec{\Sigma }}_g B^\top = \left( \begin{array}{c|c} {\varvec{\Sigma }}_g&{}{\textbf{0}}_{K\times 1}\\ \hline {\textbf{0}}_{1\times K}&{}0 \end{array}\right) . \end{aligned}$$

Therefore, the complete data (i.e., observed counts ${\textbf{W}}$ and unobserved class label indicator variable) log-likelihood using the marginal density of ${\textbf{W}}$ is

$$\begin{aligned} \ell&=\log \left[ \prod _{i=1}^{n}\prod _{g=1}^G \pi _g f_g(\textbf{w}_i\mid \vartheta _g)\right] ^{z_{ig}}=\sum _{i=1}^n\sum _{g=1}^Gz_{ig}\left\{ \log \pi _g + \log \left[ \int p({\textbf{w}}_i|{\varvec{\eta }}_i)p({\varvec{\eta }}_i|{\tilde{{\varvec{\mu }}}}_g,\tilde{{\varvec{\Sigma }}}_g)d{\varvec{\eta }}_i\right] \right\} . \end{aligned}$$

To perform variational inference on the mixture model, we substitute $\log \left[ \int p({\textbf{w}}_i|{\varvec{\eta }}_i)p({\varvec{\eta }}_i|{\varvec{\mu }}_g,{\varvec{\Sigma }}_g) d{\varvec{\eta }}_i \right]$ by the variational Gaussian lower bound ${\tilde{F}}\left( {\textbf{m}},V,\tilde{{\varvec{\mu }}},\tilde{{\varvec{\Sigma }}},\xi \right)$ derived in Section "A variational Gaussian lower bound". Hence, the variational Gaussian lower bound of complete data log likelihood can be written as:

9 $$\begin{aligned} \begin{aligned} \tilde{\mathscr {L}} =&\sum _{i=1}^n\sum _{g=1}^Gz_{ig}\log \pi _g + \sum _{i=1}^n\sum _{g=1}^Gz_{ig} {\textbf{w}}_i^\top {\textbf{m}}_{ig}-\sum _{i=1}^n\sum _{g=1}^Gz_{ig}\left( \sum _{k=1}^{K+1} w_{ik}\right) \\&\quad \left\{ \xi _i^{-1}\left[ \sum _{k=1}^{K+1}\exp \left( m_{igk}+\dfrac{ v_{igk}^2}{2}\right) \right] -1+\log (\xi _i)\right\} \\&\quad +\sum _{i=1}^n\sum _{g=1}^Gz_{ig}\left\{ \frac{1}{2}\log |B^\top \tilde{{\varvec{\Sigma }}}_g B|-\frac{1}{2}({\textbf{m}}_{ig}-\tilde{{\varvec{\mu }}}_g)^\top {\tilde{{\varvec{\Sigma }}}}_g^{*}({\textbf{m}}_{ig}-{\tilde{{\varvec{\mu }}}}_g)-\frac{1}{2}\mathop {\text {Tr}}({\tilde{{\varvec{\Sigma }}}}_g^{*} V_{ig})+\frac{1}{2}\sum _{k=1}^K\log ( v_{igk}^2)+\dfrac{K}{2}\right\} . \end{aligned} \end{aligned}$$

Hence, we need to find optimal solutions to variational parameters $({\textbf{m}}_{ig},V_{ig},\xi _i)$ that are associated with each observation ${\textbf{w}}_i, i=1,\dots ,n$, as well as the model group-specific Gaussian parameters $(\tilde{{\varvec{\mu }}}_g, \tilde{{\varvec{\Sigma }}}_g), g=1,\ldots , G$, such that the complete data variational Gaussian lower bound ${\tilde{\mathscr {L}}}$ is maximized. The use of VGA provides great reduction in the computational time.

### The variational EM algorithm

Parameter estimation can be done in an iterative EM-type approach, from here on referred to as variational EM such that the following steps are iterated until convergence. For the parameters that do not have a closed form solution to the optimization, we perform one step of Newton’s method to approximate the root to their first derivatives.

Step 1: Conditional on the variational parameters $({\textbf{m}}_{ig},V_{ig},\xi _i)$ and model group-specific Gaussian parameters $(\tilde{{\varvec{\mu }}}_g, \tilde{{\varvec{\Sigma }}}_g)$, ${\mathbb {E}}(Z_{ig}{\textbf{W}}_i)$ is computed. Given $(\tilde{{\varvec{\mu }}}_g, \tilde{{\varvec{\Sigma }}}_g)$,
  $$\begin{aligned} {\mathbb {E}}\left( Z_{ig}\mid {\textbf{w}}_i\right) =\frac{\pi _g f_g({\textbf{w}}_i\mid \tilde{{\varvec{\mu }}}_g,{\tilde{{\varvec{\Sigma }}}}_g)}{\sum _{h=1}^G\pi _h f_h({\textbf{w}}_i\mid \tilde{{\varvec{\mu }}}_h,{\tilde{{\varvec{\Sigma }}}}_h)}. \end{aligned}$$
  This involves the marginal distribution of ${\textbf{W}}$ and hence, we use an approximation of ${\mathbb {E}}\left( Z_{ig}\mid {\textbf{w}}_i\right)$ where we replace the marginal density ${\textbf{W}}$ by the exponent of ELBO such that
  $$\begin{aligned} {\hat{z}}_{ig}:=\dfrac{\pi _g \exp \left\{ {\tilde{F}}\left( {\textbf{w}}_i,{\textbf{m}}_{ig},V_{ig},\tilde{{\varvec{\mu }}}_{g},\tilde{{\varvec{\Sigma }}}_{g},\xi _i\right) \right\} }{\sum _{j=1}^{G}\pi _j \exp \left\{ {\tilde{F}}\left( {\textbf{w}}_i,{\textbf{m}}_{ij},V_{ij},\tilde{{\varvec{\mu }}}_{j},\tilde{{\varvec{\Sigma }}}_{j},\xi _i\right) \right\} }. \end{aligned}$$
Step 2: Update $${\hat{\xi }}_i,{\hat{{\textbf{m}}}}_{ig},{\hat{V}}_{ig}$$:
  update ${\hat{\xi }}_i$ according to Eq. (6);
  update ${\hat{{\textbf{m}}}}_{ig}$ by performing one step of Newton’s method for approximating the root to the derivative in Eq. (7), then let ${\hat{m}}_{ig(K+1)}=0$;
  for $k=1,\dots ,K$, update ${\hat{v}}_{igk}^2$ by performing one step of Newton’s method searching root to the derivative in Eq.  (8), let ${\hat{v}}_{ig(K+1)}^2=0$, then ${\hat{V}}_{ig} = \mathop {\text {diag}}({\hat{v}}_{ig1}^2,\dots ,{\hat{v}}_{ig(K+1)}^2)$.
Step 3: Update $\pi _{ig}$, ${\tilde{{\varvec{\mu }}}}_g$ and ${\tilde{{\varvec{\Sigma }}}}_g$ as
  $$\begin{aligned} {\hat{\pi }}_{ig}&=\frac{\sum _{n=1}^n {\hat{z}}_{ig}}{n};\quad \hat{\tilde{{\varvec{\mu }}}}_g = \dfrac{\sum _{i=1}^n {\hat{z}}_{ig}{\hat{{\textbf{m}}}}_{ig}}{\sum _{i=1}^n {\hat{z}}_{ig}}; \quad \hat{\tilde{{\varvec{\Sigma }}}}_g = \dfrac{\sum _{i=1}^n {\hat{z}}_{ig} \left[ {\hat{V}}_{ig}+({\hat{{\textbf{m}}}}_{ig}-\hat{\tilde{{\varvec{\mu }}}}_g)({\hat{{\textbf{m}}}}_{ig}-\hat{\tilde{{\varvec{\mu }}}}_g)^\top \right] }{\sum _{i=1}^n {\hat{z}}_{ig}}. \end{aligned}$$

Note that the original parameters ${\varvec{\mu }}_{g}$ and ${\varvec{\Sigma }}_{g}$ can be obtained by the transformation

$$\begin{aligned} {\hat{{\varvec{\mu }}}}_{g} = B^\top \hat{\tilde{{\varvec{\mu }}}}_g;\quad {\hat{{\varvec{\Sigma }}}}_{g} = B^\top \hat{\tilde{{\varvec{\Sigma }}}}_g B. \end{aligned}$$

An Aitken acceleration criterion^48^ is employed to stop the iterations. More specifically, at $t$th iteration, when $t >2$, calculate

$$\begin{aligned} \begin{aligned} a^{(t-1)}&= \dfrac{\ell ^{(t)}-\ell ^{(m-1)}}{\ell ^{(t-1)}-\ell ^{(t-2)}};\quad \ell _{\infty }^{(t)} = \ell ^{(t-1)}+\dfrac{1}{1-a^{(t-1)}}\left( \ell ^{(t)}-\ell ^{(t-2)}\right) , \end{aligned} \end{aligned}$$

where $\ell ^{(t)}={\tilde{F}}\left( {\textbf{w}}_i,{\textbf{m}}_{ig},V_{ig},\tilde{{\varvec{\mu }}}_{g},\tilde{{\varvec{\Sigma }}}_{g},\xi _i\right)$ is the variational Gaussian lower bound who approximates the log likelihood at $t$th iteration. Then, the algorithm will be stopped when $\left| \ell _{\infty }^{(t)}-\ell _{\infty }^{(t-1)}\right| < \varepsilon$ for a given $\varepsilon$^49^. In our analysis, $\varepsilon$ is set to be $1\times 10^{-3}$.

#### Hybrid approach

While the VGA based approach only approximates the posterior distribution and it does not guarantee exact posterior^50^, it is computationally efficient. On the other hand, a fully Bayesian MCMC based approach can generate exact results, fitting such models can take substantial computational time. For example, fitting one iteration using a fully Bayesian MCMC model for a five dimensional dataset (from Simulation study 1) with $n=1000$ takes on average of 45 minutes. In a clustering context, the number of iterations required for the analysis is typically in hundreds. Thus, we provide a computationally efficient hybrid approach in which

- Step 1: Fit the model using the VGA based approach.
- Step 2: Estimate the component indicator variable $Z_{ig}$ conditional on the parameter estimates from the VGA based approach.
- Step 3: Using the parameter estimates from Step 1 as the initial values for the parameters and using the classification from Step 2, compute the MCMC based expectation for the latent variable ${\tilde{{\varvec{\eta }}}}_{ig}$ as:
  $${\mathbb{E}}\left( {\tilde{\eta }_{{ig}} |{\mathbf{W}}_{i} } \right) \simeq \frac{1}{R}\sum\limits_{{k = 1}}^{R} {\tilde{\eta }_{{ig}}^{{(k)}} } .$$
  And $\varvec{\theta }_{ng}^{(k)}$ is a random sample from the posterior distribution of ${\tilde{{\varvec{\eta }}}}_{ig}$ simulated via the $\texttt{RStan}$ package^51^ for iterations $k= 1,\ldots , R$ (after discarding the burn-in).
- Step 4: Obtain the final estimates of the model parameters as:
  $$\begin{aligned} {\hat{\pi }}_{ig}&=\frac{\sum _{n=1}^n {\hat{z}}_{ig}}{n}; \quad \hat{\tilde{{\varvec{\mu }}}}_g = \frac{\sum _{n=1}^n {\hat{z}}_{ig}{\mathbb {E}}\big (\tilde{{\varvec{\eta }}}_{ig}\big )}{\sum _{n=1}^n {\hat{z}}_{ig}};\quad \hat{\tilde{{\varvec{\Sigma }}}}_g = \dfrac{\sum _{i=1}^n {\hat{z}}_{ig} {\mathbb {E}}\left[ (\hat{{\tilde{{\varvec{\eta }}}}}_{ig}-\hat{{\tilde{{\varvec{\mu }}}}}_g)(\hat{{\tilde{{\varvec{\eta }}}}}_{ig}-\hat{\tilde{{\varvec{\mu }}}}_g)^\top \right] }{\sum _{i=1}^n {\hat{z}}_{ig}}. \end{aligned}$$

The hybrid approach comes with a substantial reduction in computational overhead compared to a traditional MCMC based approach but it can generate samples from the exact posterior posterior distribution. Detailed comparison on computational time among the VGA based approach, the hybrid approach, and the MCMC-EM approach could be found in the results section. When the primary goal is to detect the underlying clusters (which is the case for our the real data analysis), the VGA based approach is sufficient. However, when the primarily goal is posterior inference, we recommend the hybrid approach as it can better yield an exact posterior similar to the MCMC-EM approach but is computationally efficient. For simulation studies 1 and 2 in which we show parameter recovery, we show parameter estimation using both VGA and the hybrid approach.

#### Initialization

For initialization of ${\hat{z}}_{ig}$, we used *k*-means clustering^52,53^ on the estimate of the underlying latent variable $\eta _i$ obtained by first calculating the underlying composition using ${\textbf{w}}_{i}/\sum _{k=1}^{K+1}{\textbf{w}}_{ik}$ for each observation; mapping this composition to the latent variable ${\textbf{y}}_i$ using the additive log-ratio transformation in Eq. (1), and transforming the variable to get ${\varvec{\eta }}_i$ through Eq. (2). For initializing the variational parameters for each observation ${\textbf{w}}_i$, we obtain ${\varvec{\eta }}_i$ first, same as in the ${\hat{z}}_{ig}$ initialization step. We use this calculated latent variable ${\varvec{\eta }}_i$ as initialization of ${\textbf{m}}_{ig}$. $V_{ig}$ for each *i* are initialized as $K+1$ diagonal matrix such that ${\tilde{V}}_{kk}=1$ for $k=1,\ldots ,K$ and $V_{kk}=0$ for $k=K+1$. $\xi _i$’s are initialized using 1. According to the initialization on the group label ${\hat{z}}_{ig}$, $\tilde{{\varvec{\mu }}}_{g}$ and $\tilde{{\varvec{\Sigma }}}_{g}$ are initialized as group-specific mean and covariance of ${\varvec{\eta }}_i$, respectively.

### Model selection and performance assessment

In the clustering context, the number of components *G* is unknown. Hence, one typically fits models for a large range of possible *G* and the number of clusters is then chosen *a posteriori* using a model selection criteria. The Bayesian information criterion (BIC)^54^ is one of the most popular criteria in the model-based clustering literature^32^. Here, we use an approximation to BIC defined as

$$\begin{aligned} \text {BIC} \approx -2\tilde{\mathscr {L}}+d\log (n), \end{aligned}$$

where $\tilde{\mathscr {L}}$, defined in Eq. (9), is the variational Gaussian lower bound of the complete data log likelihood, and *d* is the number of free parameters in the model. Specifically, when fitting a $G-$component model, $d=\frac{(K+1)K}{2}\times G+K\times G+G-1$.

When the true class labels are known (e.g., in simulation studies), we assess the performance of our proposed model using the adjusted Rand index (ARI)^55^. It is a measure of the pairwise agreement between the predicted and true classifications such that an $\text {ARI}$ of 1 indicates perfect classification and 0 indicates that the classification obtained is no better than by chance.

## Results

### Main simulation studies

To illustrate the performance of our proposed clustering framework, we conducted two sets of simulation studies. For both studies, the $i-$th observed counts data ${\textbf{W}}_i$ are generated as:

1. First, we generate the total counts $\sum _{k=1}^{K+1} W_{ik}$ as a random number from a uniform distribution *U*[5000, 10000].
2. Given pre-specified group specific parameters ${\varvec{\mu }}_g$ and ${\varvec{\Sigma }}_g$, we transform using Eq. (3) to get ${\tilde{{\varvec{\mu }}}}_g$ and ${\tilde{{\varvec{\Sigma }}}}_{g}$ and generate ${\varvec{\eta }}_i$ from $\mathop {\text {N}}({\tilde{{\varvec{\mu }}}}_g,{\tilde{{\varvec{\Sigma }}}}_g)$.
3. Based on ${\varvec{\eta }}_i$, we calculate ${\varvec{\Theta }}_i$ using the inverse additive log-ratio transformation $\phi ^{-1}$ using Eq. (4).
4. Using ${\varvec{\Theta }}_i$ as the underlying composition, together with the total counts generated at the first step, we generate discrete random numbers ${\textbf{W}}_i$ from multinomial distributions.
5. To initialize the variational parameters, we need to use the additive log-ratio transformation which takes the log transformation of the observed count for taxa *k* divided by total count for all taxa for sample *i*. If there are any 0 in the generated count data, we substitute the 0 with 1 for initialization.

We also compared the performance of our proposed model to Dirichlet-multinomial mixture models (DMMs)^20^ which is widely used to cluster microbiome data. Implementation of the Dirichlet mixture model is available in the R package $\texttt{DirichletMultinomial}$^56^. We also applied Gaussian mixture models (GMMs) on the ALR-transformed compositions derived from these datasets with BIC for model selection. The GMMs were fitted using the $\texttt{Mclust}$ function in the R package $\texttt{mclust}$. A family of finite mixture models with different covariance structures are implemented in $\texttt{mclust}$. The GMM model with unconstrained covariance structure “VVV” (the one that is most comparable to our proposed unrestricted covariance structure) encountered computational error for all simulated datasets. Only models assuming a spherical shape converged.

#### Simulation study 1

**Figure 1:**
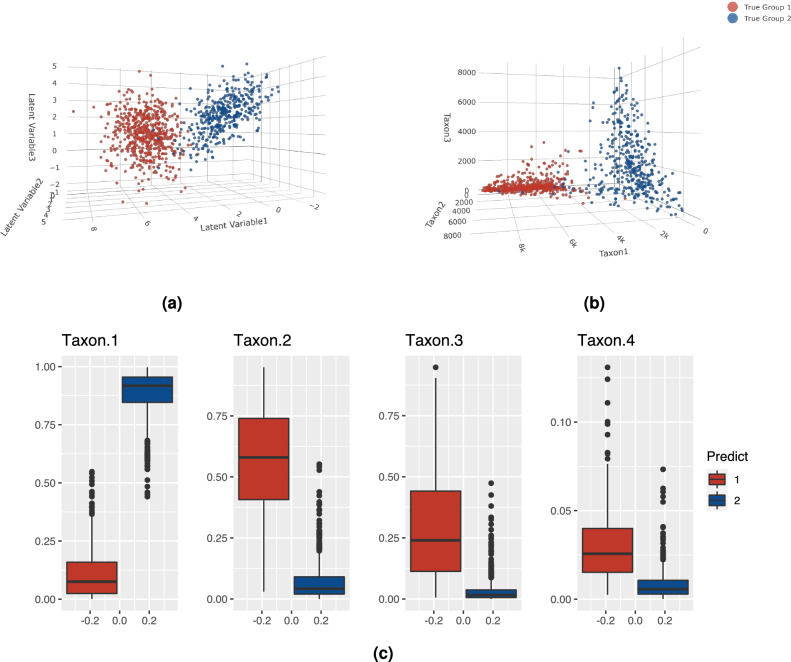
True and predicted cluster structure for one of the 100 datasets in Simulation Study 1. Panel (**a**) is the three-dimensional scatter plot of the underlying latent variable highlighted in true labels. Panel (**b**) is the first three dimensions of the observed count highlighted in true labels. Panel (**c**) is the relative abundance of observed counts of the four taxa for the predicted clusters. For this dataset, ARI was 0.95.

In this simulation study, we generated 100 datasets where the underlying latent variable ${\textbf{Y}}$ came from two-component, three-dimensional multivariate Gaussian distributions with mixing proportions ${\varvec{\pi }}= (0.6,0.4)$; see Fig. 1a. The first three dimensions of the observed counts ${\textbf{W}}$ are shown in Fig. 1b. The first component consists of $n_1=600$ observations and the second component consists of $n_2=400$ observations. The parameters used to generate the datasets are summarized in the Supplementary Table 1. We fitted the models with $G=1,\ldots ,5$ on all 100 datasets. In 100 out of 100 datasets, BIC selected a two-component model. The models selected by BIC yielded an average ARI $=0.94$ with a standard deviation of 0.02. The average and standard deviation of the estimated parameters for all 100 datasets using the VGA approach are summarized in Supplementary Table 1 and the hybrid approach are summarized in Supplementary Table 2. Note that the parameter estimations using both approaches are very close to the true value of the parameters.

The hybrid approach comes with a substantial reduction in computational overhead compared to a traditional MCMC-based approach but it can generate samples from the exact posterior distribution. The average computation time for Simulation Study 1 using the proposed VGA approach was 2.64 (sd of 0.61) minutes. The mean computation time using the hybrid approach was 47.78 (sd of 16.45) minutes. On the other hand, it took on average 45.14 (sd of 15.84) minutes for one iteration of the full Bayesian, and the number of iterations required for clustering is typically in the hundreds.

Figure 1c illustrates a clear difference in the distribution of the relative abundance of taxa in the two predicted groups. We also ran the DMMs and the GMMs on the ALR transformed compositions for $G=1:5$ and selected the best model using BIC. In all 100 out of 100 datasets, a $G=4~\text {or}~5$ model was selected for DMM with an average ARI of 0.46 (sd of 0.05). Similarly, in all 100 out of 100 datasets, a $G=4~\text {or}~5$ model was selected for the GMMs with an average ARI of 0.39 (sd of 0.03). Both the DMMs and GMMs overestimated the number of components by splitting the true clusters into multiple clusters with some misclassifications among them.

#### Simulation study 2

**Figure 2:**
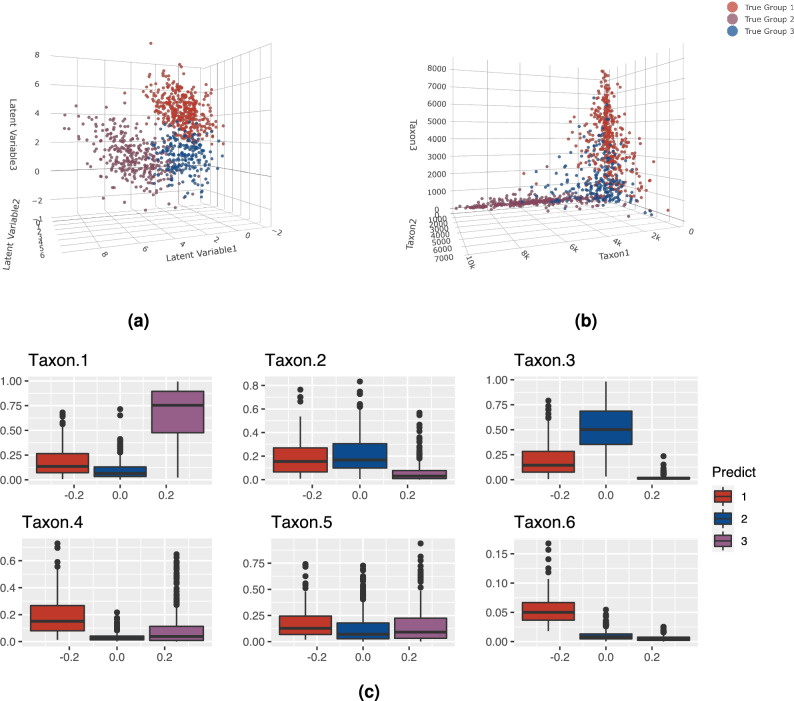
True and predicted cluster structure for one of the 100 datasets in Simulation Study 2. Panel (**a**) is the first three dimensions of the underlying latent variable highlighted in true labels. Panel (**b**) is the first three dimensions of the observed count highlighted in true labels. Panel (**c**) is the relative abundance of observed counts of the four taxa for the predicted clusters. For this dataset, ARI was 0.95.

In this simulation study, we generated 100 datasets with the underlying latent variable ${\textbf{Y}}$ from three component five-dimensional multivariate Gaussian distributions (see Fig. 2a for the three-dimensional scatter plot of the first three dimensions of the underlying latent variable ${\textbf{Y}}_i$).

There are $n_1=300$ observations in Group 1, $n_2=400$ observations in Group 1, and $n_3=200$ observations in Group 3. The true parameters are summarized in Supplementary Table 3. Figure 2b shows the first three dimensions of the observed counts ${\textbf{W}}_i$’s. There is a clearer separation between the groups when visualizing the latent variables as opposed to the observed counts.

The proposed algorithm was applied on all 100 datasets where for each dataset, we fitted the models with for $G=1,\dots ,5$. In all 100 datasets, a $G=3$ model was selected using the BIC and an overall mean ARI of 0.93 (sd of 0.02). The average and standard deviation of the estimated parameters for all 100 datasets using the VGA approach are summarized in Supplementary Table 3 and the hybrid approach are summarized in Supplementary Table 4. Note that the parameter estimation using both approaches are very close to the true value of the parameters. The average computation time for Simulation Study 2 using the proposed VGA approach was 3.03 (sd of 0.94) minutes. It took on average 40.84 (sd of 16.64) minutes for only one iteration of the MCMC-EM algorithm using the fully Bayesian approach (and the number of iterations required until convergence can be in the hundreds); whereas the mean computation time for fitting the hybrid approach until convergence (i.e., the sum of the computational times for all iterations) was 43.87 (sd of 17.58) minutes. Figure 2c illustrates a clear difference in the distribution of the relative abundance of taxa in the predicted groups. We also ran the DMMs on the observed abundance matrix and the GMMs on the ALR-transformed compositions for $G=1:5$ and selected the best model using BIC for both approaches. In all 100 out of 100 datasets, both the DMMs and the GMMs overestimated the number of components. The DMMs selected a $G=4$ model for 7 datasets and selected a $G=5$ model for the remaining 93 datasets with an average ARI of 0.31 (sd of 0.03). The GMM selected a $G=5$ model for all 100 datasets with an average ARI of 0.57 (sd of 0.03).

### Additional simulation studies

To test the performance of the proposed algorithm on higher dimensional datasets, as well as datasets generated from a mixture of Dirichlet-multinomial models, we performed a series of 10 additional simulation studies, each containing 100 datasets, as described below:

- Generate 100 datasets from a two-component mixture of logistic normal multinomial models with each of the following:
  - *K*, the dimension of the latent variable, being 5, 10,  and 20;
  - *n*, the sample size, being 100, 200,  and 500.
  - True parameters are the same for different *n* but the same *K*.
- Generate 100 datasets from a mixture of two-component Dirichlet-multinomial models with dimension 6, and a sample size of 200.
- Generate 100 datasets from a mixture of two-component high dimensional logistic normal multinomial models with K=50 and $n=500.$

We ran the proposed algorithm for $G=1:5$ on all datasets and used BIC for model selection. We also applied the DMMs and GMMs on the ALR-transformed compositions derived from these datasets with BIC for model selection.

**Table 1:** Summary of the number of times the correct model is selected along with the average ARI (with standard deviation, across all 100 datasets ) and average time per simulation (in seconds; with standard deviation) for completion for the 100 datasets for each of the 11 simulation studies described in the Additional Simulation Studies section fitting the proposed algorithm, the Dirichlet-multinomial mixture (DMM) models, and fitting the Gaussian mixture model (GMM) on additive log-ratio (ALR) transformed composition data.

Simulation setting	Proposed algorithm			DMM		GMM on ALR transformed data	
	Average time in sec. (sd)	Correct G	ARI (sd)	Correct G	ARI (sd)	Correct G	ARI (sd)
K=5, n=100	3.20 (1.46)	100	0.98 (0.03)	1	0.00 (0.01)	2	0.47 (0.10)
K=5, n=200	5.76 (1.39)	100	0.99 (0.01)	5	0.00 (0.04)	0	0.42 (0.05)
K=5, n=500	16.90 (5.26)	100	0.99 (0.01)	3	0.11 (0.06)	0	0.40 (0.02)
K=10, n=100	6.18 (1.47)	100	1.00 (0.02)	64	0.42 (0.26)	0	0.51 (0.11)
K=10, n=200	14.28 (2.03)	100	1.00 (0.00)	88	0.57 (0.10)	0	0.45 (0.06)
K=10, n=500	55.22 (10.77)	100	1.00 (0.00)	0	0.37 (0.11)	0	0.45 (0.05)
K=20, n=100	12.00 (3.72)	100	1.00 (0.01)	20	0.14 (0.29)	28	0.77 (0.18)
K=20, n=200	39.45 (11.21)	100	1.00 (0.00)	99	0.63 (0.13)	0	0.47 (0.06)
K=20, n=500	151.12 (22.52)	100	1.00 (0.00)	0	0.48 (0.10)	0	0.43 (0.03)
DMM (k=5, n=200)	11.12 (5.02)	61	0.78 (0.18)	100	0.95 (0.07)	0	0.43 (0.09)
High Dimensional - K=50, n=500	1128.73 (312.71)	100	1.00 (0.00)	29	0.50 (0.39)	71	0.88 (0.19)

When data were generated from a mixture of logistic normal multinomial models, in all simulation scenarios, the proposed algorithm identified the correct number of components for all 100 datasets with average ARI $\ge 0.98$. Also, it is observed that, in general, when sample size increases, the average ARI also increases and the standard deviation of ARI decreases. However, the Dirichlet-multinomial mixture model did not perform as well on data simulated from the logistic normal multinomial mixture models. Even in the case of $K = 20, n = 200$, where it correctly selected the two-component model 99 out of 100 times, the average ARI was only 0.63 with a standard deviation of 0.13. The GMMs on the ALR transformed data did not perform well either. While all models with different covariance structures available in the $\texttt{mclust}$ package were fitted, the most comparable one with unrestricted covariance structure, specifying $\mathtt{modelName = ``VVV''}$, encountered computational errors for all simulated datasets. Only models assuming a spherical shape converged and the GMM tended to overestimate the number of clusters (see Supplementary Table 5). Table 1 provides the number of correct G selected across 100 datasets fitting the proposed algorithm, the DMM, and the GMM on ALR transformed data, and the average ARI with standard deviation computed across all 100 datasets in each simulation scenario. Note that $G>2$ encountered computational issues when fitting GMM with unrestricted spherical cluster model (“VII”) on ALR transformed data for high dimensional $K=50$ scenario for all datasets. Thus, only $G=1$ and $G=2$ were fitted and $G=1,\ldots ,5$ could only be fitted for the model with equal spherical covariance across components (“EII”) for the GMM. In 71 out of the 100 datasets, a $G=2$ model with“VII” covariance structure was selected as the best fitting model. Although ARI here is high compared to fitting GLM on ALR-transformed data from other simulation settings, it must be noted that models with $G>2$ encountered computational issues for the “VII” covariance structure.

When the data was generated from the Dirichlet-multinomial mixture models, our proposed model was able to recover the underlying cluster in 61 out of the 100 datasets with an average ARI of 0.78 and standard deviation of 0.18 whereas the Dirichlet-multinomial mixture model was able to recover the underlying cluster structure in all 100 datasets with an average ARI of 0.95 (sd=0.07). When performing each simulation study, the computational job was distributed onto a computer cluster, where the proposed algorithm applied on each one of the 100 datasets was run on a one-core slot. Table 1 summarizes the average elapsed time for running the proposed algorithm in, with standard deviation. In most cases, it takes the proposed algorithm less than 60 seconds. As the number of observations and the dimensionality of data increases, the time to convergence increases as well.

The number of times each $G=1:5$ were selected by the proposed algorithm are summarized in Supplementary Table 5. In nine out of the ten studies, our approach was able to identify the correct number of components for all 100 datasets. We also summarized the average of $L_1$ norm between the true parameters and the estimated values along with the standard errors for the simulations with data generated from a mixture of logistic normal multinomial models in Supplementary Table 6. It shows that, when the dimensionality is low, the proposed algorithm can not only identify the correct underlying group structure but also is able to recover the true parameters well. As the dimensionality increases, the proposed algorithm can still capture the true number of components in the data with high classification accuracy and the estimated central location parameter (${\varvec{\mu }}$) is also close to the true value. However, the estimation of the spread parameter (${\varvec{\Sigma }}$) become less precise as dimensionality becomes higher; however, the distance between the true and the estimated parameters decreases as the sample size becomes larger.

### Real data analysis

#### Scenario 1: Clustering microbiome data at a lower taxonomic level

Here, we utilized our proposed algorithm to cluster the microbiome dataset at a lower taxonomic level. We applied our proposed algorithms to two previously published microbiome datasets.

- **The martínez dataset**: The study compares the fecal microbiota of individuals (40 adults) from two non-industrialized regions (20 participants from each of the Asaro and Sausi communities) in Papua New Guinea (PNG) with the individuals (22 adults) from the United States (US). The individuals from the Asaro and Sausi communities live a traditional agriculture-based lifestyle. The study found a greater bacterial diversity and lower inter-individual variations in the microbiome compositions of PNG individuals that were distinctly different from the individuals from industrialized US societies but no difference in bacterial diversity between the two PNG communities. The dataset was previously used for cluster analysis by Shi et al.^57^ and is available through the R package MicrobiomeCluster via https://github.com/YushuShi/MicrobiomeCluster.git. Here, we conducted the analysis at the OTU level.
- **The ferretti 2018 dataset**: The Ferretti 2018 study^58^ aims to understand the acquisition and development of the infant microbiome and assess the impact of the maternal microbiomes on the development of an infant’s microbial communities from birth to 4 months of life. Twenty five mother- infant pairs who vaginally delivered healthy newborns at full term were recruited for the study. For each mother, stool (a proxy for gut microbiome), dorsum tongue swabs (for oral microbiome), vaginal introitus swabs (for vaginal microbiome), intermammary cleft swabs (skin microbiome) and breast milk were obtained. Here, we applied our algorithms to a subset of the dataset to compare the oral microbiome of the infants with their mothers. Oral samples of infants were taken at two different time points: Day 1 and Day 3. Here, we used measurements from Day 1. The resulting dataset consists of 39 individuals (23 adults and 16 infants). The dataset available through the R package curatedMetagenomicData^59^ as FerrettiP_2018 dataset. We conducted the analysis at the genus level.

As our approach is currently not designed for high dimensional data, we utilize two different approaches for dimension reduction prior to clustering:

- In Case I, we first extracted the top ten most abundant taxa using the R package HMP^60^and used it for the clustering analysis. This approach requires no prior information on the cluster structure and can be utilized in a true clustering scenario. Here, we used the top ten most abundant taxa for both datasets. To preserve the compositional nature of the data, the remaining taxa were all grouped into a taxa category “Others”. This “Other” taxa was then used as the reference level for conducting the additive log-ratio transformation.
- In Case II, we first utilized the R package ALDEx2^61,62^ for differential abundance analysis on the observed taxa counts to identify the taxa that are different among different groups in the datasets. This step is analogous to conducting differential expression analysis in RNA-seq studies before performing cluster analysis to identify variables that are group differentiating.

The motivation behind proposing two different cases is to illustrate that while Case I requires no prior information on cluster structure, using the top most abundant taxa may not be always appropriate. When the top most abundant taxa contain group differentiating information, the proposed approach provides a good clustering performance. However, when the taxa with lower abundance are group differentiating features, in such case, not including those features may result in a decrease in the clustering performance. When the taxa that are group differentiating are identified in Case II using differential abundance analysis, the proposed approach can provide a better clustering performance on the same datasets.

We applied our algorithm to all datasets for $G=1$ to 4. We repeated the analysis 10 times with different *k*-means initialization and selected the final model using BIC. We also ran the Dirichlet-multinomial mixture model with the same set of taxa and the GMM on the transformed latent variable for $G=1$ to 4 on all datasets and utilized BIC for model selection. A summary of the clustering performances is provided in Table 2.

**Table 2:** Cross tabulation of the clusters obtained by our proposed algorithm and Dirichlet-multinomial mixture model on all three real datasets.

	Proposed (ARI: 1)		DMM model (ARI: 0.61)			GMM model (ARI: 0.46)			
	Estimated clusters		Estimated clusters			Estimated clusters			
	1	2	1	2	3	1	2	3	4
The Martínez Dataset									
Case I									
US	22	–	22	–	–	22	–	–	–
PNG	–	40	-	25	15	–	11	14	15

	Proposed (ARI: 1)		DMM model (ARI: 0.76)			GMM model (ARI: 0.69)			
	Estimated clusters		Estimated clusters			Estimated clusters			
	1	2	1	2	3	1	2	3	4
Case II									
US	22	–	22	–	–	12	10	–	–
PNG	–	40	–	33	7	–	–	35	5

	Proposed (ARI: 0.71)		DMM model (ARI: 0.62)			GMM model (ARI: 0.61)			
	Estimated clusters		Estimated clusters			Estimated clusters			
	1	2	1	2		1	2	3	4
The Ferretti Oral microbiome subset									
Case I									
Infant	13	3	12	4		14	1	1	–
Adult	–	23	-	23		–	18	2	3

	Proposed (ARI: 1)		DMM model (ARI: 0.80)			GMM model (ARI: 0.37)			
	Estimated clusters		Estimated clusters			Estimated clusters			
	1	2	1	2		1	2	3	4
Case II									
Infant	16	–	14	2		7	3	6	–
Adult	–	23	–	23		–	–	7	16

For both datasets, our approach outperformed the Dirichlet-multinomial mixture models and the GMM applied to the latent variables under both scenarios. When there is a good overlap between the group differentiating taxa and most abundant (i.e., in the case of the Martínez dataset), the proposed approach, Dirichlet multinomial mixture model, and the Gaussian mixture model with the transformed variables all provide good clustering performance for Case I and Case II. On the other hand, when the group differentiating taxa differ from the most abundant taxa (i.e., in the case of the Ferretti dataset), in such scenario, all models tested perform better for Case II.

#### Scenario 2: clustering healthy microbiome samples from Human microbiome project at a higher taxonomic level

We also applied our algorithm to the HMP2012 dataset^63^ available from the R package curatedMetagenomicData^59^. The dataset comprises microbiome compositions of 129 males and 113 females. “Healthy” individuals (i.e., individuals without any evidence of diseases) were recruited and samples were collected from one or more of the five different body sites (nasal cavity, oral cavity, skin, stool, and vagina). In total, we have $n=748$ microbiome sample profiles. Here, we focused on analyzing the dataset at the Phylum level. First, the top ten most abundant phyla were extracted and phyla with at least 5% non-zero counts were retained which resulted in 8 phyla that were retained. The remaining phyla were all grouped into a phylum category “Others” which was used as a reference level for conducting log-ratio transformation.

**Figure 3:**
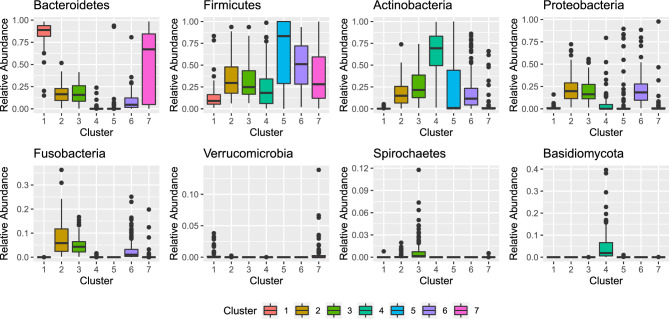
Boxplot of relative abundances of the top most abundant phyla for all seven components.

We utilized our algorithm to the HMP2012 dataset for $G=1$ to 10 and we repeated the analysis 10 times with different *k*-means initialization and selected the final model using BIC. A seven-component LNM-MM was selected. Figure 3 provides a visualization of the relative abundances of the top most abundant phyla across the seven components and the cross-tabulation of the estimated cluster membership against the five body sites is provided in Table 3.

**Table 3:** Cross tabulation of the estimated cluster membership against the five body sites and the compositions of the estimated clusters.

Clusters	Nasal cavity	Oral cavity	Skin	Stool	Vagina
1	–	–	–	73	–
2	–	124	–	–	–
3	–	116	–	–	–
4	59	2	26	–	1
5	29	6	1	3	39
6	3	160	–	1	–
7	2	6	–	70	27

**Figure 4:**
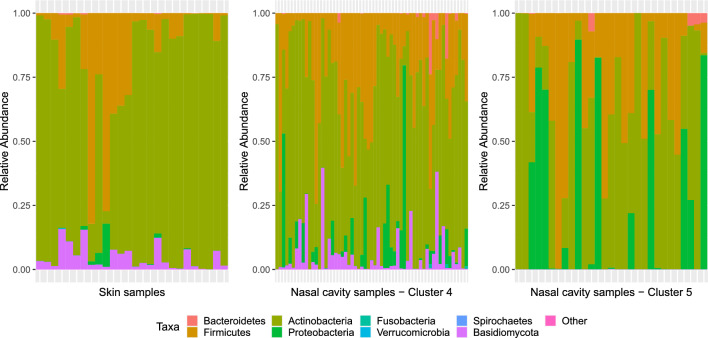
Visualization of the relative abundances of skin samples and samples from nasal cavity assigned to clusters 4 and 5.

Cluster 1 comprised only of stool samples and clusters 2 and 3 comprised only oral cavity samples. Cluster 6 is also comprised primarily of oral cavity samples. Cluster 4 comprised a mix of samples primarily from the nasal cavity and skin; cluster 5 comprised a mix of samples primarily from the nasal cavity and vagina; and cluster 7 comprised a mix of samples primarily of the stool and vagina. It is interesting to note that samples from the same body sites are clustered into multiple clusters, in some cases, with samples from other body sites. For example, samples from the nasal cavity were clustered into two clusters: cluster 4 and cluster 5 where the cluster 5 also comprised samples from the skin. Visualization of the relative abundance of samples from the skin and nasal cavity assigned to clusters 4 and 5 in Fig. 4 reveals that in fact, the microbiome profiles of samples from the nasal cavity in cluster 4 are more similar to the microbiome profiles of samples from the skin than to the samples from the nasal cavity assigned to cluster 5. This is in alignment with the findings of the original study^63^ where a high overlap between the nasal cavity samples and skin samples is observed in the principal coordinates plot of the samples.

Similarly, samples from the oral cavity were assigned to three clusters: 2, 3 and 6. Boxplots of the relative abundances of the samples in Fig. 3 reveal that the relative abundances of taxa in the three clusters are different. Samples in cluster 3 have a much higher relative abundance of Spirochaetes compared to clusters 2 and 3 and samples in cluster 6 have a higher relative abundance of Firmicutes compared to samples in clusters 2 and 3 and a lower relative abundance of Fusobacteria compared to samples in cluster 2. The DMM model and the GMM applied on latent variable were also fitted to the same dataset for $G=1$ to 10 and BIC was utilized for model selection. A six-component model was selected by DMM and a similar trend as our proposed algorithm was observed. The samples from the oral cavity were assigned to distinct three clusters, and part of the samples from the nasal cavity were clustered together with samples from the skin. Similar to what we observed from the simulation studies and the other real data results, GMM on the ALR transformed data overestimated the number of components here as well. A ten-component model was selected and a similar trend as the proposed algorithm result was observed, where the two oral cavity sample clusters and one of the stool sample clusters were further split into two smaller clusters each separately.

## Discussion

A model-based clustering framework for microbiome compositional data is developed using a mixture of logistic normal multinomial models. The novelty of this work is multi-fold. Previous work^23^ has indicated that the logistic normal multinomial models can model the dependency of the bacterial composition in a microbiome compositional data in a more flexible way than the commonly used Dirichlet-multinomial models. The latent variables in the logistic normal multinomial model are assumed to follow a multivariate Gaussian distribution and a closed form expression of the log-likelihood or posterior distributions of the latent variables do not exist. Hence, prior work on model fitting relied on Markov chain Monte Carlo (MCMC) sampling techniques that come with a heavy computational burden. This is compounded in the clustering context where MCMC sampling needs to be utilized at every iteration of the variant of the EM algorithm that is typically utilized for parameter estimation. Here, we employed a variational Gaussian approximation to the posterior distribution of the latent variable and implemented a generalized EM algorithm that does not rely on MCMC sampling thus making it feasible to extend these models for clustering. This also opens up the possibility of efficiently scaling and extending these models to a high-dimensional setting.

Through simulation studies, we have shown that the proposed algorithm delivers accurate parameter recovery and good clustering performance. The proposed method is also illustrated on three real datasets in Section "Real data analysis" where we demonstrate that the proposed models can recover the interesting cluster (group) structure in the real data. While in the datasets with small sample sizes, we focus on small dimensional data by data aggregation to most differentially abundant genera in real data analysis, for larger datasets, more taxa can be used. Because of adopting an underlying Gaussian distribution, the number of parameters in the covariance matrix alone grows quadratically with *K*. Thus, in high dimensional datasets with small sample size, estimating $\varvec{\Sigma }^{-1}$ becomes more challenging as it can lead to degenerate solutions and a host of other issues related to model convergence and fitting while using a traditional maximum likelihood-based expectation-maximization approach. This a well-known issue with Gaussian mixture models and is typically dealt with either variable/feature selection or dimension reduction. Feature selection typically eliminates the redundant or irrelevant variables and reduces computational cost, provides a better understanding of data and improves predictions^64^. ALDEx2 utilized here is a widely used variable/feature selection technique specifically designed for microbiome data that identifies taxa that are differentially abundant in different conditions. Through a comparative study of ALDEx2 with other approaches commonly used for differential abundance analysis, Quinn et al.^65^ showed that ALDEx2 has high precision (i.e., few false positives) across different scenarios. However, information on the group structure or conditions may not be available a-priori. In such cases, one may conduct feature selection by selecting the top few most abundant taxa and collapsing low-abundant taxa into one category “Others” to preserve the compositional nature of the data. Alternately, mixtures of logistic multinomial models can be extended to high-dimensional data by introducing subspace clustering techniques through the latent variable^66–68^. This will be the topic of some future work. Additionally, it has been well-established that different environmental or biological covariates can affect the microbiome compositions. Some future work will also focus on developing a mixture of logistic normal multinomial regression models to investigate the relationship of biological/environmental covariates with the microbiome compositions within each cluster.

### Supplementary Information

Supplementary Information.

## Data Availability

The datasets used in this manuscript are publicly available from the R package curatedMetagenomicData (https://bioconductor.org/packages/curatedMetagenomicData/) and MicrobiomeCluster (https://github.com/YushuShi/MicrobiomeCluster).
