# Supplementary material for: Clustering microbiome data using mixtures of logistic normal multinomial models
Source: Sci Rep. 2023 Sep 7;13:14758. doi: 10.1038/s41598-023-41318-8 (PMC10484970; doi:10.1038/s41598-023-41318-8)
Supplement: Supplementary file 1 — Supplementary Information. [file 41598_2023_41318_MOESM1_ESM.pdf]

Supplementary material for "Clustering microbiome data using mixtures of logistic normal multinomial models"

Yuan Fang[1] Sanjeena Subedi[2,*]

[1]School of Pharmacy and Pharmaceutical Sciences, Binghamton University, State University of New York, 4400 Vestal Parkway East, Binghamton, NY 13902, USA.
[2]School of Mathematics & Statistics, 4302 Herzberg Laboratories, Carleton University, 1125 Colonel By Drive, Ottawa, Ontario, K1S 5B6, Canada.
*Correspondence to: sanjeena.dang@carleton.ca.

1 Mathematical Detail

Consider the following transformed parameter $\boldsymbol{\eta}$ from $\mathbf{Y}$:

$$\boldsymbol{\eta} = B\mathbf{Y}, \quad \text{where } B = \begin{pmatrix} 1 & 0 & \dots & 0 \\ 0 & 1 & \dots & 0 \\ \vdots & \vdots & \dots & \vdots \\ 0 & 0 & \dots & 1 \\ 0 & 0 & \dots & 0 \end{pmatrix},$$

is a $(K+1) \times K$ matrix which takes the form as an identity matrix attached by a row of K zeros. Given the assumption that the true distribution of $\mathbf{Y}$ is $\mathrm{N}(\boldsymbol{\mu}, \boldsymbol{\Sigma})$, we have the true distribution of $\boldsymbol{\eta}$ to be Gaussian too, with mean $\tilde{\boldsymbol{\mu}}$ and covariance matrix $\tilde{\boldsymbol{\Sigma}}$, where

$$\tilde{\boldsymbol{\mu}} = B\boldsymbol{\mu} = (\boldsymbol{\mu}, 0)^\top; \tilde{\boldsymbol{\Sigma}} = B\boldsymbol{\Sigma}B^\top = \left(\begin{array}{c|c} \boldsymbol{\Sigma} & \mathbf{0}_{K\times 1} \\ \hline \mathbf{0}_{1\times K} & 0 \end{array}\right).$$

For computational convenience, we further assume that V has a diagonal structure, with each diagonal element denoted as v_k^2 such that

$$v_k^2 = \begin{cases} v_k^2, & k = 1, \dots, K \\ 0, & k = K+1. \end{cases}$$

We also denote the $k-$th element of $\mathbf{m}$ as m_k such that

$$m_k = \begin{cases} m_k, & k = 1, \dots, K \\ 0, & k = K+1. \end{cases}$$

Recall that we have the following decomposition of the ELBO

$$F(q(\boldsymbol{\eta}), \mathbf{w}) = F(\mathbf{m}, V) = -\int q(\boldsymbol{\eta}) \log q(\boldsymbol{\eta}) d\boldsymbol{\eta} + \int q(\boldsymbol{\eta}) \log p(\boldsymbol{\eta}) d\boldsymbol{\eta} + \int q(\boldsymbol{\eta}) \log p(\mathbf{w}|\boldsymbol{\eta}) d\boldsymbol{\eta};$$

among which, the first integral by definition is the entropy of the variational Gaussian distribution $q(\boldsymbol{\eta}|\mathbf{m}, V)$:

$$-\int q(\boldsymbol{\eta})\log q(\boldsymbol{\eta})d\boldsymbol{\eta} = -\mathbb{E}_{q(\boldsymbol{\eta}|\mathbf{m},V)}\left(q(\boldsymbol{\eta})\right) = \frac{1}{2}\sum_{k=1}^{K}\log(v_k^2) + \frac{K}{2}\log(2\pi) + \frac{K}{2}.$$

The second integral can be evaluated explicitly as well, which turn into the expected value of the log density function of $p(\boldsymbol{\eta}) = \mathrm{N}(\boldsymbol{\eta}|\tilde{\boldsymbol{\mu}}, \tilde{\boldsymbol{\Sigma}})$ with respect to $q(\boldsymbol{\eta}|\mathbf{m}, V)$:

$$\begin{aligned}\int q(\boldsymbol{\eta})\log p(\boldsymbol{\eta})d\boldsymbol{\eta} &= \mathbb{E}_{q(\boldsymbol{\eta}|\mathbf{m},V)}\left(\log p(\boldsymbol{\eta})\right)\\ &= \mathbb{E}_{q(\boldsymbol{\eta}|\mathbf{m},V)}\left(-\frac{K}{2}\log(2\pi) - \frac{1}{2}\log|\tilde{\boldsymbol{\Sigma}}| - \frac{1}{2}(\boldsymbol{\eta}-\boldsymbol{\mu})^\top\tilde{\boldsymbol{\Sigma}}^{-1}(\boldsymbol{\eta}-\boldsymbol{\mu})\right)\\ &= -\frac{K}{2}\log(2\pi) - \frac{1}{2}\log|\tilde{\boldsymbol{\Sigma}}| - \frac{1}{2}(\mathbf{m}-\tilde{\boldsymbol{\mu}})^\top\tilde{\boldsymbol{\Sigma}}^{-1}(\mathbf{m}-\tilde{\boldsymbol{\mu}}) - \frac{1}{2}\mathrm{Tr}(\tilde{\boldsymbol{\Sigma}}^{-1}V).\end{aligned}$$

Due to the special structure of $\tilde{\boldsymbol{\Sigma}}$, we have $|\tilde{\boldsymbol{\Sigma}}| = 0$ and $\tilde{\boldsymbol{\Sigma}}^{-1}$ does not exist, which brings in a computational issue. Therefore, we substitute $|\tilde{\boldsymbol{\Sigma}}|$ by $|\boldsymbol{\Sigma}| = |B^\top\tilde{\boldsymbol{\Sigma}}B|$ and $\tilde{\boldsymbol{\Sigma}}^{-1}$ by the generalized inverse of $\tilde{\boldsymbol{\Sigma}}$

$$\tilde{\boldsymbol{\Sigma}}^* = \left(\begin{array}{c|c}\boldsymbol{\Sigma}^{-1} & \mathbf{0}_{K\times 1}\\ \hline \mathbf{0}_{1\times K} & 0\end{array}\right).$$

Hence we have

$$\begin{aligned}\int q(\boldsymbol{\eta})\log p(\boldsymbol{\eta})d\boldsymbol{\eta} &= -\frac{K}{2}\log(2\pi) - \frac{1}{2}\log|\boldsymbol{\Sigma}| - \frac{1}{2}(\mathbf{m}-\tilde{\boldsymbol{\mu}})^\top\tilde{\boldsymbol{\Sigma}}^*(\mathbf{m}-\tilde{\boldsymbol{\mu}}) - \frac{1}{2}\mathrm{Tr}(\tilde{\boldsymbol{\Sigma}}^*V)\\ &= -\frac{K}{2}\log(2\pi) - \frac{1}{2}\log|B^\top\tilde{\boldsymbol{\Sigma}}B| - \frac{1}{2}(\mathbf{m}-\tilde{\boldsymbol{\mu}})^\top\tilde{\boldsymbol{\Sigma}}^*(\mathbf{m}-\tilde{\boldsymbol{\mu}}) - \frac{1}{2}\mathrm{Tr}(\tilde{\boldsymbol{\Sigma}}^*V).\end{aligned}$$

The third integral is intractable, because of the log-sum exponential term. We upper bound this term with a Taylor expansion similar to previous literature (Blei and Lafferty (2007)) resulting in the following

$$\begin{aligned}\mathbb{E}_{q(\boldsymbol{\eta}|\mathbf{m},V)}\left[\log\left(\sum_{k=1}^{K+1}\exp\eta_k\right)\right] &\le \xi^{-1}\left\{\sum_{k=1}^{K+1}\mathbb{E}_{q(\boldsymbol{\eta}|\mathbf{m},V)}\left[\exp(\eta_k)\right]\right\} - 1 + \log(\xi)\\ &= \xi^{-1}\left\{\sum_{k=1}^{K+1}\exp\left(m_k + \frac{v_k^2}{2}\right)\right\} - 1 + \log(\xi).\end{aligned}$$

Therefore, the third integral is lower bounded by

$$\begin{aligned}\int q(\boldsymbol{\eta})\log p(\mathbf{w}|\boldsymbol{\eta})d\boldsymbol{\eta} &= \mathbb{E}_{q(\boldsymbol{\eta}|\mathbf{m},V)}\left[\mathbf{w}^\top\boldsymbol{\eta} - \sum_{k=1}^{K+1}w_k\log\left(\sum_{k=1}^{K+1}\exp\eta_k\right)\right]\\ &= \mathbf{w}^\top\mathbf{m} - \left(\sum_{k=1}^{K+1}w_k\right)\mathbb{E}_{q(\boldsymbol{\eta}|\mathbf{m},V)}\left[\log\left(\sum_{k=1}^{K+1}\exp\eta_k\right)\right]\\ &\ge \mathbf{w}^\top\mathbf{m} - \left(\sum_{k=1}^{K+1}w_k\right)\left\{\xi^{-1}\left[\sum_{k=1}^{K+1}\exp\left(m_k + \frac{v_k^2}{2}\right)\right] - 1 + \log(\xi)\right\}.\end{aligned}$$

Combining all three integrals, we obtain a concave variational Gaussian lower bound to the model evidence

$$\begin{aligned}\tilde{F}\left(\mathbf{m}, V, \tilde{\boldsymbol{\mu}}, \tilde{\boldsymbol{\Sigma}}, \xi\right) = &\mathbf{w}^\top \mathbf{m} - \left(\sum_{k=1}^{K+1} w_k\right)\left\{\xi^{-1}\left[\sum_{k=1}^{K+1} \exp\left(m_k + \frac{v_k^2}{2}\right)\right] - 1 + \log(\xi)\right\} \\ &- \frac{1}{2}\log|B^\top \tilde{\boldsymbol{\Sigma}} B| - \frac{1}{2}(\mathbf{m} - \tilde{\boldsymbol{\mu}})^\top \tilde{\boldsymbol{\Sigma}}^*(\mathbf{m} - \tilde{\boldsymbol{\mu}}) - \frac{1}{2}\operatorname{Tr}(\tilde{\boldsymbol{\Sigma}}^* V) \\ &+ \frac{1}{2}\sum_{k=1}^{K}\log(v_k^2) + \frac{K}{2}.\end{aligned} \tag{1}$$

We maximize this lower bound with respect to the variational parameters $\xi, \mathbf{m}, V$.

First, we maximize the lower bound 1 with respect to ξ. The derivative with respect to ξ is

$$\frac{\partial \tilde{F}}{\partial \xi} = \left(\sum_{k=1}^{K+1} w_k\right)\left\{-\xi^{-2}\left[\sum_{k=1}^{K+1} \exp\left(m_k + \frac{v_k^2}{2}\right)\right] + \xi^{-1}\right\},$$

which yields an optimizer at

$$\hat{\xi} = \sum_{k=1}^{K+1} \exp\left(m_k + \frac{v_k^2}{2}\right).$$

Second, we maximize with respect to $\mathbf{m}$, of which the derivative is given as

$$\frac{\partial \tilde{F}}{\partial \mathbf{m}} = \mathbf{w} - \tilde{\boldsymbol{\Sigma}}^*(\mathbf{m} - \tilde{\boldsymbol{\mu}}) - \left(\sum_{k=1}^{K+1} \mathbf{w}_k\right)\xi^{-1}\exp\left(\mathbf{m} + \frac{\mathbf{v}^2}{2}\right),$$

with $\mathbf{v}^2 = (v_1^2, \ldots, v_K^2, 0)$ denoting the diagonal element of V as a vector. There is no analytical solution to this derivative and so we use Newton's method to approximate the root to this derivative, with a constrain that the $(K+1)-$th element is zero. The procedure requires the Hessian matrix with respect to $\mathbf{m}$:

$$H_{\mathbf{m}} = -\tilde{\boldsymbol{\Sigma}}^* - \left(\sum_{k=1}^{K+1} \mathbf{w}_k\right)\xi^{-1}\operatorname{diag}\left\{\exp\left(\mathbf{m} + \frac{\mathbf{v}^2}{2}\right)\right\}.$$

Finally, we optimize with respect to v_k, for $k = 1, \ldots, K$ and always set v_{K+1} as zero. Again, there are no analytical solutions and Newton's method is used for each coordinate. The first and second derivatives with respect to v_k for $k = 1, \ldots, K$ are given as follows

$$\begin{aligned}\frac{\partial \tilde{F}}{\partial v_k} &= v_k^{-1} - v_k \tilde{\boldsymbol{\Sigma}}^*_{k,k} - \left(\sum_{k=1}^{K+1} \mathbf{w}_k\right)\xi^{-1}\exp\left(m_k + \frac{v_k^2}{2}\right) v_k; \\ \frac{\partial^2 \tilde{F}}{\partial v_k^2} &= -v_k^{-2} - \left(\sum_{k=1}^{K+1} \mathbf{w}_k\right)\xi^{-1}\exp\left(m_k + \frac{v_k^2}{2}\right)\left(v_k^2 + 1\right).\end{aligned}$$

At each iteration of the variational EM algorithm, when we maximize the variational Gaussian lower bound $\tilde{F}(\mathbf{m}, V, \tilde{\boldsymbol{\mu}}, \tilde{\boldsymbol{\Sigma}}, \xi)$ with respect to the variational parameter set $(\xi, \mathbf{m}, V)$, we take one step of update based on the optimization discussed above.

2 Parameter recovery of Simulation Studies

Table 1: True and estimated parameters along with the standard deviations from the one hundred datasets from Simulation Study 1 using VGA approach; average ARI= 0.94 (0.02).

	**Component 1** ($n = 600$)	
**Parameter**	**True**	**Average of the estimates (sd)**
$\boldsymbol{\mu}$	$[5, \quad 2, \quad 1]$	$[5.00\ (0.05), \quad 2.01\ (0.05), \quad 1.01\ (0.04)]$
$\boldsymbol{\Sigma}$	$\begin{bmatrix} 1 & 0.4 & 0 \\ 0.4 & 1.2 & -0.5 \\ 0 & -0.5 & 1 \end{bmatrix}$	$\begin{bmatrix} 1.05\ (0.07) & 0.44\ (0.06) & 0.02\ (0.05) \\ 0.44\ (0.06) & 1.24\ (0.08) & -0.48\ (0.06) \\ 0.02\ (0.05) & -0.48\ (0.06) & 1.00\ (0.06) \end{bmatrix}$
	**Component 2** ($n = 400$)	
**Parameter**	**True**	**Average of the estimates (sd)**
$\boldsymbol{\mu}$	$[1, \quad 3, \quad 2]$	$[1.01\ (0.07), \quad 3.01\ (0.05), \quad 2.01\ (0.05)]$
$\boldsymbol{\Sigma}$	$\begin{bmatrix} 1.4 & 0.2 & -0.65 \\ 0.2 & 1 & 0 \\ -0.65 & 0 & 1 \end{bmatrix}$	$\begin{bmatrix} 1.39\ (0.11) & 0.21\ (0.07) & -0.63\ (0.08) \\ 0.21\ (0.07) & 1.00\ (0.08) & -0.01\ (0.05) \\ -0.63\ (0.08) & -0.01\ (0.05) & 0.96\ (0.08) \end{bmatrix}$

Table 2: True and estimated parameters along with the standard deviations from the one hundred datasets for the latent variable parameters in Simulation Study 1 using the hybrid approach.

	**Component 1** ($n = 600$)	
**Parameter**	**True**	**Average of the estimates (sd)**
$\boldsymbol{\mu}$	$[5, \quad 2, \quad 1]$	$[4.99(0.05), \quad 2.00(0.05), \quad 0.99(0.04)]$
$\boldsymbol{\Sigma}$	$\begin{bmatrix} 1 & 0.4 & 0 \\ 0.4 & 1.2 & -0.5 \\ 0 & -0.5 & 1 \end{bmatrix}$	$\begin{bmatrix} 0.98(0.07) & 0.41(0.05) & 0.00(0.05) \\ 0.41(0.05) & 1.21(0.08) & -0.50(0.06) \\ 0.00(0.05) & -0.50(0.06) & 0.99(0.06) \end{bmatrix}$
	**Component 2** ($n = 400$)	
**Parameter**	**True**	**Average of the estimates (sd)**
$\boldsymbol{\mu}$	$[1, \quad 3, \quad 2]$	$[0.98(0.07), \quad 3.01(0.05), \quad 2.02(0.05)]$
$\boldsymbol{\Sigma}$	$\begin{bmatrix} 1.4 & 0.2 & -0.65 \\ 0.2 & 1 & 0 \\ -0.65 & 0 & 1 \end{bmatrix}$	$\begin{bmatrix} 1.32(0.11) & 0.23(0.07) & -0.59(0.07) \\ 0.23(0.07) & 1.00(0.08) & -0.01(0.05) \\ -0.59(0.07) & -0.01(0.05) & 0.95(0.08) \end{bmatrix}$

Table 3: True and estimated values for the parameters from Simulation Study 2 using VGA approach; Average ARI= 0.93 (*sd* 0.02).

	**Component 1** ($n = 300$)	
**Parameter**	**True**	**Estimated (sd)**
$\boldsymbol{\mu}$	[5, 2, 1, 2, 3]	[5.02 (0.09), 2.02 (0.06), 1.01 (0.07), 2.01 (0.07), 3.02(0.06)]
$\boldsymbol{\Sigma}$	$\begin{bmatrix} 2 & -0.2 & 0.8 & -1 & 0 \\ -0.2 & 1 & -0.2 & 0 & -0.4 \\ 0.8 & -0.2 & 1.4 & 0.6 & 0 \\ -1 & 0 & 0.6 & 1.6 & 0.2 \\ 0 & -0.4 & 0 & 0.2 & 1.2 \end{bmatrix}$	$\begin{bmatrix} 2.06\ (0.17) & -0.14\ (0.09) & 0.85\ (0.13) & -0.96\ (0.12) & 0.06\ (0.10) \\ -0.14\ (0.09) & 1.01\ (0.09) & -0.15\ (0.07) & 0.01\ (0.08) & -0.36\ (0.07) \\ 0.85\ (0.13) & -0.15\ (0.07) & 1.46\ (0.13) & 0.64\ (0.10) & 0.04\ (0.09) \\ -0.96\ (0.12) & 0.01\ (0.08) & 0.64\ (0.10) & 1.64\ (0.14) & 0.22\ (0.08) \\ 0.06\ (0.10) & -0.36\ (0.07) & 0.04\ (0.09) & 0.22\ (0.08) & 1.25\ (0.11) \end{bmatrix}$
	**Component 2** ($n = 400$)	
**Parameter**	**True**	**Estimated (sd)**
$\boldsymbol{\mu}$	[2, 3, 4, 1, 2]	[2.04 (0.07), 3.04 (0.06), 4.04 (0.06), 1.02 (0.06), 2.02 (0.07)]
$\boldsymbol{\Sigma}$	$\begin{bmatrix} 1.4 & 0.65 & 0.4 & 0 & 0 \\ 0.65 & 1 & 0.2 & 0 & 0.4 \\ 0.4 & 0.2 & 1 & 0.6 & 0 \\ 0 & 0 & 0.6 & 1.2 & 0.8 \\ 0 & 0.4 & 0 & 0.8 & 2 \end{bmatrix}$	$\begin{bmatrix} 1.34\ (0.10) & 0.61\ (0.06) & 0.36\ (0.08) & -0.02\ (0.08) & -0.02\ (0.09) \\ 0.61\ (0.06) & 0.96\ (0.08) & 0.16\ (0.06) & -0.02\ (0.06) & 0.39\ (0.08) \\ 0.36\ (0.08) & 0.16\ (0.06) & 0.96\ (0.08) & 0.59\ (0.06) & -0.02\ (0.08) \\ -0.02\ (0.08) & -0.02\ (0.06) & 0.59\ (0.06) & 1.21\ (0.08) & 0.80\ (0.09) \\ -0.02\ (0.09) & 0.39\ (0.08) & -0.02\ (0.08) & 0.80\ (0.09) & 2.01\ (0.15) \end{bmatrix}$
	**Component 3** ($n = 200$)	
**Parameter**	**True**	**Estimated (sd)**
$\boldsymbol{\mu}$	[1, 1, 1, 1, 1]	[0.99 (0.08), 1.03 (0.08), 1.10 (0.10), 0.96 (0.09), 1.03 (0.09)]
$\boldsymbol{\Sigma}$	$\begin{bmatrix} 1 & 0 & 0 & 0 & 0 \\ 0 & 1 & 0 & 0 & 0 \\ 0 & 0 & 1 & 0 & 0 \\ 0 & 0 & 0 & 1 & 0 \\ 0 & 0 & 0 & 0 & 1 \end{bmatrix}$	$\begin{bmatrix} 0.99\ (0.12) & -0.00\ (0.08) & -0.03\ (0.08) & -0.01\ (0.08) & -0.03\ (0.06) \\ 0.00\ (0.08) & 1.00\ (0.11) & 0.06\ (0.10) & -0.03\ (0.08) & 0.03\ (0.08) \\ -0.03\ (0.08) & 0.06\ (0.10) & 1.11\ (0.14) & -0.05\ (0.08) & 0.03\ (0.09) \\ -0.01\ (0.08) & -0.03\ (0.08) & -0.05\ (0.08) & 1.00\ (0.11) & 0.00\ (0.07) \\ -0.03\ (0.06) & 0.03\ (0.08) & 0.03\ (0.09) & 0.00\ (0.07) & 1.00\ (0.10) \end{bmatrix}$

Table 4: True and estimated parameters (mean (sd)) for Simulation Study 2 using the hybrid approach.

	**Component 1** ($n = 300$)	
**Parameter**	**True**	**Estimated (sd)**
$\boldsymbol{\mu}$	[5, 2, 1, 2, 3]	[5.00(0.09), 2.00(0.06), 0.99(0.08), 2.00(0.08), 3.00(0.06)]
$\boldsymbol{\Sigma}$	$\begin{bmatrix} 2 & -0.2 & 0.8 & -1 & 0 \\ -0.2 & 1 & -0.2 & 0 & -0.4 \\ 0.8 & -0.2 & 1.4 & 0.6 & 0 \\ -1 & 0 & 0.6 & 1.6 & 0.2 \\ 0 & -0.4 & 0 & 0.2 & 1.2 \end{bmatrix}$	$\begin{bmatrix} 2.01(0.17) & -0.18(0.08) & 0.80(0.13) & -0.99(0.11) & 0.01(0.10) \\ -0.18(0.08) & 0.98(0.08) & -0.19(0.07) & 0.00(0.08) & -0.39(0.07) \\ 0.80(0.13) & -0.19(0.07) & 1.40(0.12) & 0.61(0.09) & 0.00(0.08) \\ -0.99(0.11) & 0.00(0.08) & 0.61(0.09) & 1.61(0.13) & 0.20(0.07) \\ 0.01(0.10) & -0.39(0.07) & 0.00(0.08) & 0.20(0.07) & 1.22(0.10) \end{bmatrix}$
	**Component 2** ($n = 400$)	
**Parameter**	**True**	**Estimated (sd)**
$\boldsymbol{\mu}$	[2, 3, 4, 1, 2]	[1.99(0.07), 2.99(0.06), 3.99(0.06), 1.00(0.06), 2.00(0.07)]
$\boldsymbol{\Sigma}$	$\begin{bmatrix} 1.4 & 0.65 & 0.4 & 0 & 0 \\ 0.65 & 1 & 0.2 & 0 & 0.4 \\ 0.4 & 0.2 & 1 & 0.6 & 0 \\ 0 & 0 & 0.6 & 1.2 & 0.8 \\ 0 & 0.4 & 0 & 0.8 & 2 \end{bmatrix}$	$\begin{bmatrix} 1.36(0.11) & 0.62(0.07) & 0.38(0.09) & 0.00(0.08) & -0.01(0.09) \\ 0.62(0.07) & 0.98(0.08) & 0.18(0.07) & -0.01(0.06) & 0.39(0.08) \\ 0.38(0.09) & 0.18(0.07) & 0.98(0.09) & 0.60(0.07) & -0.01(0.08) \\ 0.00(0.08) & -0.01(0.06) & 0.60(0.07) & 1.20(0.08) & 0.79(0.09) \\ -0.01(0.09) & 0.39(0.08) & -0.01(0.08) & 0.79(0.09) & 1.98(0.15) \end{bmatrix}$
	**Component 3** ($n = 200$)	
**Parameter**	**True**	**Estimated (sd)**
$\boldsymbol{\mu}$	[1, 1, 1, 1, 1]	[0.99(0.08), 0.96(0.08), 0.97(0.09), 1.01(0.09), 1.01(0.09)]
$\boldsymbol{\Sigma}$	$\begin{bmatrix} 1 & 0 & 0 & 0 & 0 \\ 0 & 1 & 0 & 0 & 0 \\ 0 & 0 & 1 & 0 & 0 \\ 0 & 0 & 0 & 1 & 0 \\ 0 & 0 & 0 & 0 & 1 \end{bmatrix}$	$\begin{bmatrix} 0.99(0.13) & -0.01(0.08) & -0.01(0.08) & -0.02(0.08) & -0.02(0.07) \\ -0.01(0.08) & 0.96(0.11) & -0.04(0.09) & 0.02(0.08) & -0.01(0.07) \\ -0.01(0.08) & -0.04(0.09) & 0.91(0.11) & 0.02(0.07) & 0.00(0.07) \\ -0.02(0.08) & 0.02(0.08) & 0.02(0.07) & 0.98(0.11) & 0.00(0.07) \\ -0.02(0.07) & -0.01(0.07) & 0.00(0.07) & 0.00(0.07) & 0.97(0.10) \end{bmatrix}$

Table 5: Summary of the number of times various G are selected for simulation studies described in the Additional Simulation Studies section.

	**Proposed algorithm**				**Dirichlet-multinomial mixture model**					**GMM on ALR transformed data**				
**Simulation setting**	$G=1$	$G=2$	$G=3$	$G=4$	$G=1$	$G=2$	$G=3$	$G=4$	$G=5$	$G=1$	$G=2$	$G=3$	$G=4$	$G=5$
K=5, n=100		100			99	1					2	6	36	56
K=5, n=200		100			94	5		1					11	89
K=5, n=500		100			1	3	40	26	30					100
K=10, n=100		100			26	64	10					7	31	62
K=10, n=200		100				88	10	1	1				1	99
K=10, n=500		100				0		17	83				4	96
K=20, n=100		100			80	20					28	43	20	9
K=20, n=200		100				99						1	20	79
K=20, n=500		100				0	30	34	36					100
DMM (k=5, n=200)	1	61	29	8	1	100							7	93
High dimensional K=50, n=500		100			27	30	17	17	9	29	71*			

*Note that $G > 2$ encountered computational issues when fitting GMM with unrestricted spherical cluster model ("VII") on ALR transformed data for high dimensional $K = 50$ scenario for all datasets. Thus, only $G = 1$ and $G = 2$ were fitted and $G = 1, \ldots, 5$ could only be fitted for the model with equal spherical covariance across components ("EII").

Table 6: Average L_1 norm with standard error of the true parameters and estimated values for simulation studies described in the Additional Simulation Studies section.

	**Component 1** ($\pi_1 = 0.5$)		
**Simulation setting**	**Average (sd) of** $\hat{\pi}_1$	**Average (sd) of** $\lvert\hat{\boldsymbol{\mu}}_1 - \boldsymbol{\mu}_1\rvert_{L_1}$	**Average (sd) of** $\lvert\hat{\boldsymbol{\Sigma}}_1 - \boldsymbol{\Sigma}_1\rvert_{L_1}$
K=5, n=100	0.4996 (0.009)	0.3789 (0.1535)	1.281 (0.3175)
K=5, n=200	0.5005 (0.0038)	0.2425 (0.1021)	0.8694 (0.2536)
K=5, n=500	0.4999 (0.0023)	0.1489 (0.0592)	0.574 (0.1597)
K=10, n=100	0.4896 (0.0129)	0.9975 (0.2892)	10.1231 (1.8573)
K=10, n=200	0.496 (0.0067)	0.7483 (0.2515)	7.1662 (1.2323)
K=10, n=500	0.5005 (0.0024)	0.588 (0.1657)	7.8635 (2.0106)
K=20, n=100	0.4546 (0.0212)	1.7992 (0.3343)	26.4108 (4.9824)
K=20, n=200	0.4991 (0.0047)	1.111 (0.266)	15.718 (1.5242)
K=20, n=500	0.4998 (6e-04)	0.7108 (0.1562)	10.1909 (0.9694)

	**Component 2** ($\pi_2 = 0.5$)		
**Simulation setting**	**Average (sd) of** $\hat{\pi}_2$	**Average (sd) of** $\lvert\hat{\boldsymbol{\mu}}_2 - \boldsymbol{\mu}_2\rvert_{L_1}$	**Average (sd) of** $\lvert\hat{\boldsymbol{\Sigma}}_2 - \boldsymbol{\Sigma}_2\rvert_{L_1}$
K=5, n=100	0.5004 (0.009)	0.3094 (0.1344)	0.9111 (0.2978)
K=5, n=200	0.4995 (0.0038)	0.203 (0.0812)	0.6462 (0.2292)
K=5, n=500	0.5001 (0.0023)	0.1293 (0.0604)	0.3882 (0.1385)
K=10, n=100	0.5104 (0.0129)	0.59 (0.1926)	3.5965 (2.1616)
K=10, n=200	0.504 (0.0067)	0.3837 (0.1212)	2.0346 (0.9415)
K=10, n=500	0.4995 (0.0024)	0.5144 (0.1382)	5.9088 (1.0227)
K=20, n=100	0.5454 (0.0212)	2.3534 (0.6456)	43.53 (12.8879)
K=20, n=200	0.5009 (0.0047)	0.8151 (0.2012)	9.4582 (2.157)
K=20, n=500	0.5002 (6e-04)	0.5151 (0.1226)	5.7646 (0.5573)
